# Near Infrared Light‐Activatable Platelet‐Mimicking NIR‐II NO Nano‐Prodrug for Precise Atherosclerosis Theranostics

**DOI:** 10.1002/advs.202304994

**Published:** 2023-11-30

**Authors:** Yun Chai, Lina Shangguan, Hui Yu, Ye Sun, Xiaoyan Huang, Yanyan Zhu, Hai‐Yan Wang, Yi Liu

**Affiliations:** ^1^ State Key Laboratory of Natural Medicines, School of Engineering China Pharmaceutical University Nanjing 211198 China; ^2^ School of Mechanical Engineering Southeast University Nanjing 211189 China

**Keywords:** atherosclerosis, nano‐prodrug, NIR‐II, nitric oxide, platelet‐mimicking

## Abstract

Atherosclerosis is a chronic inflammatory disease that affects arteries and is the main cause of cardiovascular disease. Atherosclerotic plaque formation is usually asymptomatic and does not manifest until the occurrence of clinical events. Therefore, early diagnosis and treatment of atherosclerotic plaques is particularly important. Here, a series of NIR‐II fluorescent dyes (RBT‐NH) are developed for three photoresponsive NO prodrugs (RBT‐NO), which can be controllably triggered by 808 nm laser to release NO and turn on the NIR‐II emission in the clinical medicine “therapeutic window”. Notably, RBT3‐NO is selected for its exhibited high NO releasing efficiency and superior fluorescence signal enhancement. Subsequently, a platelet‐mimicking nano‐prodrug system (RBT3‐NO‐PEG@PM) is constructed by DSPE‐mPEG_5k_ and platelet membrane (PM) for effectively targeted diagnosis and therapy of atherosclerosis in mice. The results indicate that this platelet‐mimicking NO nano‐prodrug system can reduce the accumulation of lipids at the sites of atherosclerotic plaques, improve the inflammatory response at the lesion sites, and promote endothelial cell migration, thereby slowing the progression of plaques.

## Graphical abstract

An activatable platelet‐mimicking NO nano‐prodrug system (RBT3‐NO‐PEG@PM) is developed for targeted NIR‐II imaging and treatment of atherosclerosis, which can be used to accurately locate atherosclerotic plaques and control the release of NO. The results show that the system can reduce the accumulation of lipid in atherosclerotic plaques, improve the inflammatory reaction in the lesion, and promote endothelial cell migration.

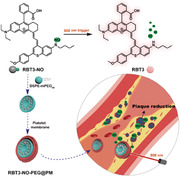

## 1 Introduction

Atherosclerosis (AS) is a chronic inflammatory disease that affects arteries and is the main cause of cardiovascular disease. At the onset of atherosclerosis, the endothelial layer is harmed, and oxidized low‐density lipoprotein (LDL) accumulates, facilitating inflammation of body tissues.^[^ ^1^ ^]^ As atherosclerotic plaques sustain to accumulate, they can obstruct blood circulatory flow, thereby inducing ischemic vascular diseases such as heart attack or stroke and so on. Currently, the main methods of clinical treatment of AS are drug therapy and endovascular intervention therapy.^[^ ^2^ ^]^ Due to the lack of targeting, the efficacy of drug treatment is difficult to determine. On the other hand, antiproliferative drugs have been shown to have a negative impact on re‐endothelialization, leading to a higher risk of thrombosis.^[^ ^3^ ^]^ Endovascular interventions may lead to the development of complications such as thrombosis or myocardial infarction.^[^ ^4^ ^]^ Moreover, atherosclerotic plaque formation is often asymptomatic and does not manifest until the occurrence of clinical events. Therefore, it is especially important to develop more efficacious methods for the early diagnosis and treatment of atherosclerotic plaques.

The rapid progress of cardiovascular nanomedicine has brought new diagnosis and treatment strategies for AS. In recent decades, all kinds of nanomaterials have been endowed with different functional characteristics, such as targeting, stimulus‐response delivery systems, imaging contrast probes, and therapeutic agents.^[^ ^5^ ^]^ The microenvironment of atherosclerotic plaques is a complex biochemical environment. Nanomaterials with catalytic, optical, and thermal properties have led to different treatment strategies for atherosclerotic plaques, such as antioxidant therapy,^[^ ^6^ ^]^ photodynamic therapy (PDT), and photothermal therapy (PTT).^[^ ^7^ ^]^ Nitric oxide (NO) was the first endogenous gaseous transmitter confirmed to regulate physiological and pathological processes.^[^ ^8^ ^]^ As a cell signaling molecule, NO can regulate vascular tone through cyclic guanosine monophosphate (cGMP)‐mediated endothelium‐dependent vasodilation. In this context, NO may act as a motive force and therapeutic agent to facilitate endothelial repair at plaque sites.^[^ ^9^ ^]^ The challenge associated with this strategy is the instability and short half‐life of NO because it can rapidly combine with hemoglobin. Therefore, to facilitate treatment, NO prodrugs are used to alleviate the lack of endothelium‐derived NO. NO nano‐prodrugs have been used to replace NO gas as a major source for biological research and to release NO via various external stimuli, such as pH,^[^ ^10^ ^]^ enzymes,^[^ ^11^ ^]^ ultrasound,^[^ ^12^ ^]^ photothermal treatment,^[^ ^13^ ^]^ or light,^[^ ^14^ ^]^ among others. We assume that this flexible and controllable NO nano‐prodrug system could provide targeted therapy to the atherosclerotic plaque sites to achieve the effect of optimized drug therapy.

Currently, kinds of imaging strategies are available for clinical atherosclerotic plaque diagnosis. Due to great advantages in detection safety and spatiotemporal resolution, noninvasive optical imaging has been extensively used for the visualization of atherosclerosis, such as near‐infrared (NIR) fluorescence imaging,^[^ ^15^ ^]^ dual/triple photon imaging,^[^ ^16^ ^]^ photoacoustic imaging (PAI),^[^ ^17^ ^]^ and upconversion luminescence imaging.^[^ ^18^ ^]^ However, the strong absorption of endogenous substances in living organisms, tissue scattering, autofluorescence background, and other interference limit the achievable resolution and depth of fluorescence imaging. Over the past decade, the development of second near‐infrared (NIR‐II, 1000–1700 nm) imaging has opened an effective avenue for high‐resolution fluorescence imaging of deep tissues due to its longer emission wavelength, lower tissue absorption, scattering, and weak autofluorescence.^[^ ^19^ ^]^

In addition, due to nonspecific uptake during systemic circulation, the NO prodrug delivery system may be scattered throughout the body, leading to significantly lower concentrations at disease sites. Platelets, as a type of blood cell, contain various components on their membrane surface, such as glycoprotein receptors and cell adhesion molecules. Considering their specific localization to macrophage‐derived foam cells, early atherosclerotic plaques and major components, platelets can be effectively used for the accurate localization of atherosclerotic plaques.^[^ ^20^ ^]^ Targeted drug use can particularly improve the accumulation ability in the lesion location, effectively improve targeted therapy for atherosclerosis, and minimize side effects.

**Scheme 1:**
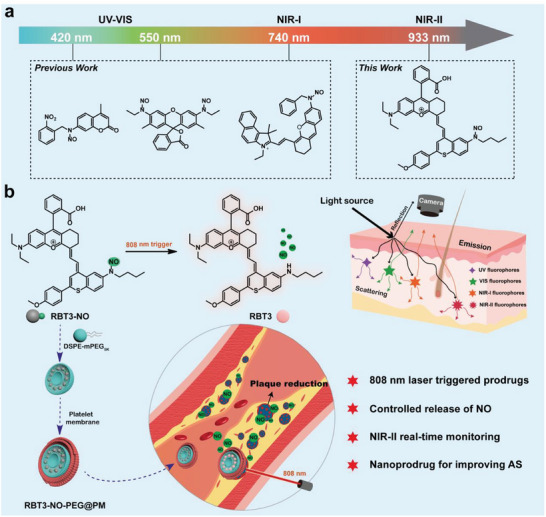
a) Comparison of emission wavelengths of several light triggered NO donors. b) Schematic diagram of nano‐prodrug RBT3‐NO‐PEG@PM for in vivo phototriggering treatment of atherosclerosis and allows simultaneous NIR‐II real‐time drug delivery monitoring.

Based on the above, it would be challenging to develop an activatable platelet‐mimicking NO nano‐prodrug delivery system for the diagnosis and treatment of atherosclerosis. In this work, a series of NIR‐II fluorescent dyes (RBT‐NH) were developed with good photostability and large Stokes shift. Importantly, with the change in the R group, this study applied RBT‐NH to construct three photoresponsive NO prodrugs (RBT‐NO) in the clinical medicine “therapeutic window”. They could be controllably triggered to release NO by 808 nm laser and generate real‐time changes in the NIR‐II spectral for monitoring prodrug delivery and evaluating NO release efficiency. Notably, RBT3‐NO exhibited higher NO release productivity and more significant fluorescence signal enhancement. To further enhance the biocompatibility level of RBT3‐NO, a NO nanomaterial (RBT3‐NO‐PEG) was prepared with DSPE‐mPEG_5k_. Subsequently, a platelet‐mimicking NO nano‐prodrug system (RBT3‐NO‐PEG@PM) was prepared by encapsulating platelet membrane (PM) for targeted diagnosis and treatment of atherosclerosis in mice (**Scheme** **1**). The results showed that the release of NO by laser triggering can reduce the accumulation of lipids at the spot of atherosclerotic plaques, improve the inflammatory response at the lesion site, and promote endothelial cell migration, thereby slowing the progression of plaques.

## 2 Results and Discussion

### 2.1 Design and Synthesis

Compared to previous work, the emission wavelength of NO donors using photolysis is in the ultraviolet visible or NIR‐I region, making the monitoring of their fluorescence signals susceptible to interference from the background of biological spontaneous fluorescence.^[^ ^21^ ^]^ On the other hand, the short wavelength of the excitation light used to excite NO donors makes it difficult for them to penetrate deeper tissues when used in organisms, making it difficult to achieve effective photolysis and better treatment. To overcome these difficulties, developing NO donors with longer excitation and emission wavelengths will be a necessary choice (Scheme 1a).

**Scheme 2:**
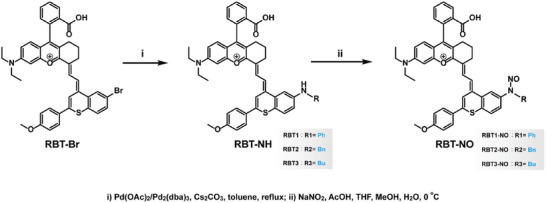
Synthetic route of RBT‐NH and RBT‐NO.

In this paper, based on the structure of rhodamine, benzothiopyrylium heterocycles were introduced as donors to expand its conjugation structure and construct an intramolecular charge transfer (ICT) mechanism, so that the highest occupied molecular orbital (HOMO) and the lowest unoccupied molecular orbital (LUMO) distributions in the excited state undergo electron transfer, which results in a large Stokes shift. In addition, a series of fluorescent dyes (RBT‐NH) have been synthesized by modifying the electron density of the donor R group to change its photophysical properties such as the molar extinction coefficient and fluorescence quantum yield. Subsequently, RBT‐NH prepare NO prodrugs (RBT‐NO) via a nitration reaction, enabling controllable light‐responsive NO gas therapy (**Scheme** **2**).

### 2.2 Spectral Characteristics

**Figure 1:**
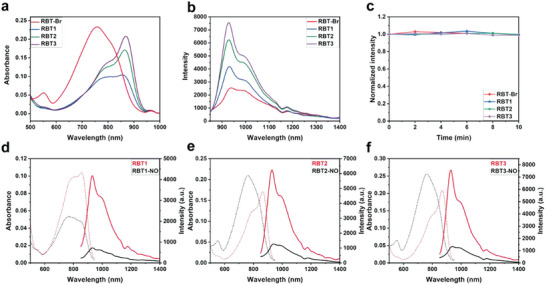
Spectral characteristics of RBT‐NH and RBT‐NO. a) Comparison of ultraviolet absorption spectra of RBT‐NH (10 µm) in dichloromethane. b) Comparison of fluorescence emission spectra of RBT‐NH (10 µm) in dichloromethane, λ_ex_ = 808 nm. c) Photostability comparison of all fluorophores (10 µm) in dichloromethane under continuous‐wave laser exposure (808 nm) at a fluence rate of 1.4 W cm^−2^, λ_ex_ = 808 nm. d–f) Ultraviolet absorption/fluorescence emission spectra of RBT‐NH and RBT‐NO (10 µm) in dichloromethane, λ_ex_ = 808 nm.

As a proof of concept, in this study we designed and synthesized a series of fluorescent dyes (RBT‐NH). Compared with the rhodamine base structure, the benzothiopyrylium heterocycle donor helps to extend the absorption/emission wavelength to reach the NIR‐II emission region, and the maximum emission wavelength is ≈930 nm (**Figure** **1**a,b). Furthermore, the introduction of n‐butylamine, which is more capable of donating electrons, onto a led to an obvious improvement in the photophysical properties of the fluorophore (Table S1, Supporting Information). For example, RBT3 exhibits a higher molar extinction coefficient and fluorescence quantum yield than RBT‐Br, resulting in its higher brightness, and the fluorescence quantum yield of RBT3 (0.106%) is ≈2 times that of RBT‐Br (0.063%). In addition, all RBT‐NH exhibited good photostability, showing one kind of anti‐quenching ability in continuous fluorescence imaging (Figure 1c). In addition, as expected, once the N‐H bond of RBT‐NH is converted into N‐nitroso by nitrosation, the absorption peak of RBT‐NH is blueshifted and the fluorescence is quenched (Figure 1d–f).

### 2.3 Photoresponsive Release Characters of Prodrugs RBT‐NO

**Figure 2:**
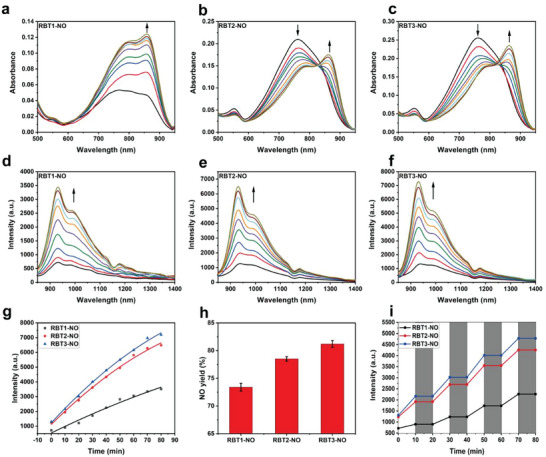
Photoresponsive release properties of prodrugs RBT‐NO. a–c) Ultraviolet absorption spectrum changes of RBT‐NO (10 µm, a) RBT1‐NO, b) RBT2‐NO, c) RBT3‐NO) in dichloromethane irradiated by 808 nm laser at a fluence rate of 0.5 W cm^−2^, time = 0, 10, 20, 30, 40, 50, 60, 70, 80 min. d–f) Fluorescence emission spectrum changes of RBT‐NO (10 µm, d) RBT1‐NO, e) RBT2‐NO, f) RBT3‐NO) in dichloromethane irradiated by 808 nm laser at a fluence rate of 0.5 W cm^−2^, time = 0, 10, 20, 30, 40, 50, 60, 70, 80 min, λ_ex_ = 808 nm. g) Comparison of fluorescence emission spectrum changes of RBT‐NO (10 µM) in dichloromethane, λ_ex_ = 808 nm. h) Comparison of NO yield of RBT‐NO (50 µm). i) Controllability of NO release from RBT‐NO (10 µm) by switching 808 nm laser “on” (white) and “off” (grey).

The prodrug solution was irradiated with an 808 nm laser as a trigger to evaluate the photouncaging efficiency of the prodrugs (RBT‐NO). As shown in **Figure** **2**, the absorption signal of RBT1‐NO at 869 nm was elevated, and the fluorescence intensity was gradually enhanced at 928 nm (Figure 2a,d). The absorption signal of RBT2‐NO at 761 nm decreased, that at 870 nm increased, and the fluorescence intensity gradually increased at 929 nm (Figure 2b,e). The absorption signal of RBT3‐NO at 762 nm decreased, the absorption signal at 872 nm increased, and the fluorescence intensity gradually increased at 933 nm (Figure 2c,f), indicating that the prodrugs RBT‐NO were decomposed into the corresponding fluorescent dyes through the light response. Due to its higher photosensitivity, RBT3‐NO has better NO release efficiency, which is due to its higher molar extinction coefficient. On the other hand, RBT3‐NO shows more obvious changes in fluorescence enhancement signals (Figure 2g), which indicates that RBT3‐NO may be a better NO donor. In addition, the photolysis rate is also an important factor in evaluating the prodrugs RBT‐NO. Using the Griess reagent system to monitor NO release (Figure S1, Supporting Information),^[^ ^22^ ^]^ the NO yields of RBT1‐NO, RBT2‐NO and RBT3‐NO are 73.4%, 78.5%, and 81.2%, respectively (Figure 2h). To further verify the controlled photolysis of the prodrugs RBT‐NO, RBT‐NO were alternately exposed by laser in the dark. As shown in the Figure 2i, obvious fluorescence signal enhancement was observed under light conditions, while the fluorescence intensity remained almost unchanged in the dark, which fully verified the controllable light response characteristics of the prodrugs RBT‐NO.

### 2.4 Photoresponsive Splitting Mechanism of RBT‐NO

**Figure 3:**
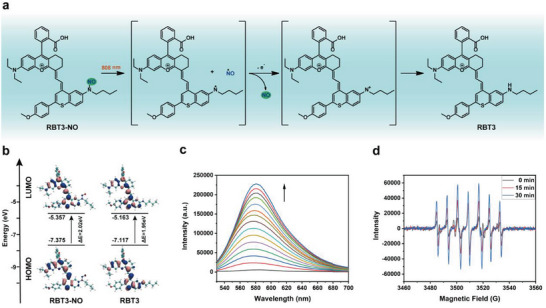
a) Photolysis splitting mechanism of RBT3‐NO. b) Energy calculation of RBT3‐NO and RBT3. c) Fluorescence emission spectrum changes of RBT3‐NO (10 µm) with RhBs (10 µm) in methanol/water (v/v, 1:1) irradiated by 808 nm laser at a fluence rate of 0.5 W cm^−2^. d) EPR spectra of spin‐trapped NO released from RBT3‐NO.

The photoresponsive splitting mechanism of RBT‐NO is shown in **Figure** **3**. The N‐nitrosamine in the structure of the prodrug RBT3‐NO undergoes the process of active intermediates with positively charged divalent nitrogen atoms when it releases NO under photolysis conditions (Figure 3a).^[^ ^23^ ^]^ To clarify the mechanism of photophysical changes of NO produced by the photolysis of prodrug RBT3‐NO, quantitative calculations were carried out using the Gaussian 16 suite of programs. The structures of the molecules were fully optimized at the B3LYP‐D3BJ/def2‐SVP level of theory. According to density functional theory (DFT) calculations, for RBT3, π electrons on HOMO are spread to the rhodamine core structure and the benzothiopyrylium heterocycles. However, no obvious electron redistribution is observed in RBT3‐NO, which coincides with the intramolecular charge transfer (ICT) effect. In addition, RBT3 exhibits a smaller energy gap (1.95 eV) than RBT3‐NO (2.02 eV), which is consistent with the redshift of maximum absorption and emission after RBT3‐NO photolysis.

To further verify the process of NO release from the prodrugs RBT‐NO under laser irradiation, the photolysis of the prodrugs RBT‐NO was verified by high performance liquid chromatography (HPLC). The retention time of RBT3‐NO was 21.35 min (Figure S4, Supporting Information). Under laser irradiation, the peak value corresponding to the retention time gradually decreased. At the same time, new peaks appeared and gradually increased. The retention time corresponding to the new peak was consistent with the retention time of 22.55 min for RBT3. Subsequently, RBT1‐NO and RBT2‐NO obtained similar results through HPLC monitoring (Figures S2 and S3, Supporting Information). In addition, the generation of RBT3 was also verified by high resolution mass spectrometry (HRMS) analysis. The corresponding value of the new peak of the prodrug RBT3‐NO under laser irradiation was m/z 723.3239, which was consistent with RBT3 (Figure S7, Supporting Information). Similar results were also obtained for RBT1‐NO and RBT2‐NO (Figures S5 and S6, Supporting Information). Next, we verified the production of NO gas. RhBs was used as a fluorescent indicator to verify the presence of NO. Once the probe contacts NO, it can open the spiral ring, convert to rhodamine B and turn on the fluorescence signal.^[^ ^24^ ^]^ As planned, when the RBT3‐NO prodrug solution with RhBs was irradiated with an 808 nm laser, fluorescence signal enhancement was monitored at 585 nm (Figure 3c). RBT1‐NO and RBT2‐NO also obtained similar results (Figure S8, Supporting Information). In addition, electron paramagnetic resonance (EPR) spectra of RBT3‐NO were carried out using 2‐phenyl‐4,4,5,5‐tetramethylimidazoline‐1‐oxyl 3‐oxide (PTIO radical), which showed that the PTI radical was generated after the PTIO radical was mediated by NO (Figure 3d). These results indicate that the photoresponsive prodrugs RBT‐NO release NO successfully.

### 2.5 Preparation and Characterization of NO Nano‐Prodrug System

**Figure 4:**
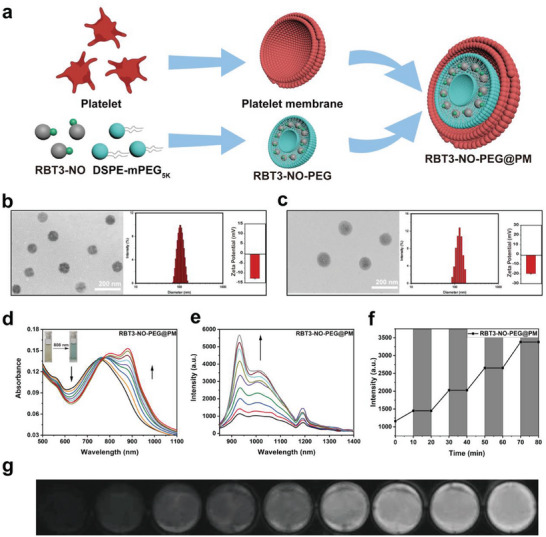
a) Preparation of RBT3‐NO‐PEG@PM. b) TEM image, DLS and Zeta potential of RBT3‐NO‐PEG. c) TEM image, DLS and Zeta potential of RBT3‐NO‐PEG@PM. d) Ultraviolet absorption spectrum changes of RBT3‐NO‐PEG@PM (10 µm, calculated as RBT3‐NO) in aqueous solution irradiated by 808 nm laser at a fluence rate of 0.5 W cm^−2^, time = 0, 10, 20, 30, 40, 50, 60, 70, 80 min. e) Fluorescence emission spectrum changes of RBT3‐NO‐PEG@PM (10 µm) in aqueous solution irradiated by 808 nm laser at a fluence rate of 0.5 W cm^−2^, time = 0, 10, 20, 30, 40, 50, 60, 70, 80 min, λ_ex_ = 808 nm. f) Controllability of NO release from RBT3‐NO‐PEG@PM (10 µm) in aqueous solution by switching 808 nm laser “on” (white) and “off” (grey). g) NIR‐II fluorescence imaging of RBT3‐NO‐PEG@PM (10 µm) in aqueous solution irradiated by 808 nm laser at a fluence rate of 0.5 W cm^−2^, time = 0, 10, 20, 30, 40, 50, 60, 70, 80 min. Excitation wavelength: 808 nm. Emitted signals were collected through passing 1000 nm LP filter.

This study selected RBT3‐NO, an excellent NO donor, as the research object. In our study, the nanoparticle coated by platelet membrane was designed and synthesized for targeting atherosclerotic plaque sites and NO gas therapy at focal sites. Platelets are tightly relevant to the development of atherosclerotic plaques, and the specific binding of the platelet membrane to plaque‐infiltrating macrophages to identify early plaques may enable in vivo follow‐up indicator therapy for early atherosclerotic plaques. Prodrug RBT3‐NO was first wrapped up in DSPE‐mPEG_5k_ micelles and then coated with platelet membrane to form the nanoparticle, and the preparation process of nanoparticles is shown in **Figure** **4**a. The prodrug RBT3‐NO is encapsulated in the hydrophobic cavity of the micelle, and the hydrophilic end of DSPE‐mPEG_5k_ is evenly dispersed on the surface of the cluster, which greatly improves the water solubility and stability of the prodrug RBT3‐NO. Transmission electron microscopy (TEM) and dynamic light scattering (DLS) indicated that RBT3‐NO‐PEG formed spherical nanostructures with a size of ≈80 nm and a hydrated particle size of ≈105 nm (Figure 4b). The zeta potential of the RBT3‐NO‐PEG was −12.6 mV. After further application of platelet membrane coating, its size and hydration particle size increased by ≈20–25 nm (Figure 4c), and the zeta potential of the RBT3‐NO‐PEG@PM was −19.9 mV, which verified the successful implementation of the film coating.

In addition, UV and fluorescence spectra confirmed that RBT3‐NO‐PEG@PM maintained good light response performance. Under the irradiation of 808 nm laser, the absorption signal of RBT3‐NO‐PEG@PM at 625 nm decreased, the absorption signal at 883 nm increased, and the fluorescence intensity gradually increased at 933 nm (Figure 4d,e). Compared with the fluorescence emission spectrum changes of RBT3‐NO, it still maintains an excellent NO release efficiency (Figure S9, Supporting Information). After photolysis, the solution also changed from light green to light yellow. At the same time, to further verify the controlled photolysis of the prodrug RBT3‐NO‐PEG@PM, laser exposure was carried out alternately in the dark. Under light conditions, obvious fluorescence signal enhancement was observed, while the fluorescence intensity remained almost unchanged in the dark (Figure 4f). In addition, the same results were detected by imaging with a porous plate containing nano‐prodrug solution, and the fluorescence intensity gradually increased after irradiation at different times. This result fully verifies the controllable optical response characteristics of RBT3‐NO‐PEG@PM (Figure 4g; Figure S10, Supporting Information).

### 2.6 Intracellular Imaging of RBT3‐NO‐PEG@PM in Living Cells

**Figure 5:**
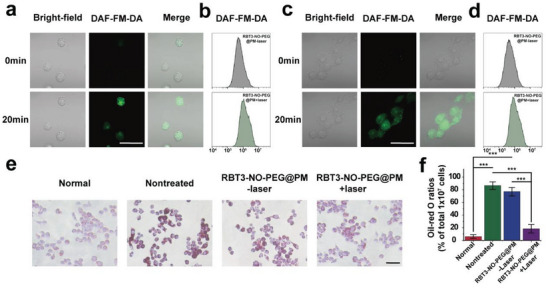
a) Fluorescence imaging and flow cytometry analysis b) of proving gaseous NO release in RAW cells co‐incubated with RBT3‐NO‐PEG@PM (15 µg mL^−1^, calculated as RBT3‐NO‐PEG) and NO indicator DAF‐FM‐DA (20 µm) with laser exposure (808 nm, 0.5 W cm^−2^), scale bar: 50 µm. c) Fluorescence imaging and flow cytometry analysis d) of proving gaseous NO release in HUVECs cells co‐incubated with RBT3‐NO‐PEG@PM (15 µg mL^−1^) and NO indicator DAF‐FM‐DA (20 µm) with laser exposure (808 nm, 0.5 W cm^−2^), scale bar: 50 µm. e) The influence of NO on lipid storage in foam cells determined with Oil Red O staining, scale bar: 50 µm. f) The ratio of Oil Red O stained cells in total 1 × 10^7^ cells. The results are showed as mean ± SD (*n* = 3). Statistical significance was assessed applying one‐way ANOVA. ^*^ *p* < 0.05, ^**^ *p* < 0.01, ^***^ *p* < 0.001.

After successfully verifying the controllable photoluminescence ability of RBT3‐NO‐PEG@PM in vitro, we verified its intracellular photolysis activity at the cellular level. First, the cytotoxicity of the nano‐prodrug RBT3‐NO‐PEG@PM was verified by the MTT method. It was observed that >80% of the cells survived under the incubation condition of 0–25 µg mL^−1^ RBT3‐NO‐PEG@PM, which revealed the low toxicity and good biocompatibility of RBT3‐NO‐PEG@PM (Figure S11, Supporting Information). Next, this study used DAF‐FM‐DA green fluorescent probe as NO catcher to verify the intracellular NO release ability of RBT3‐NO‐PEG@PM through fluorescence imaging.^[^ ^25^ ^]^ As shown in **Figure** **5** and Figure S12 (Supporting Information), the green fluorescence of both RAW cells and HUVECs cells was gradually enhanced, indicating that NO released by RBT3‐NO‐PEG@PM reacted with DAF‐FM‐DA, which verified the ability of nanoparticles to control light release by NO at the cellular level. Consistent with this, flow cytometry analysis further confirmed the successful release of the nano‐prodrug within cells under laser irradiation (Figure 5b,d).

### 2.7 The Role of RBT3‐NO‐PEG@PM in Improving Lipid Accumulation in Cells

Having confirmed the gas controlled photorelease of RBT3‐NO‐PEG@PM in the cell, we set out to verify the ability of NO gas to improve lipid accumulation at the cellular level. Atherosclerosis, as the name implies, is a disease of lipid deposition in the arterial endothelium, which leads to the proliferation of the arterial endothelium and endothelial cell damage. NO has an excellent function of relaxing vascular smooth muscle, which can scale up blood flow, guard against fat and other sediments from adhering to the vascular wall, thus suppressing the thickening of blood vessel walls, and decreasing the probability of vascular blockage. NO can also effectively inhibit platelet adhesion, prevent its migration and aggregation to the subendothelial layer, inhibit the expression of thrombus protein tissue factor that initiates the endogenous clotting pathway on the surface of endothelial cells, facilitate the migration and proliferation of endothelial cells, and improve lipid accumulation. As shown in Figure S13 (Supporting Information), in the RBT3‐NO‐PEG@PM group, compared with the control group, the promotion of HUVECs cell mobility increased by 46%, indicating that NO can promote the migration of endothelial cells and exert its ability to improve atherosclerosis at the cellular level. As shown in Figure 5e, the oil red O staining results of foam cells showed that lipid accumulation was significantly improved in the RBT3‐NO‐PEG@PM light group, further supporting the above results.

### 2.8 Evaluation of Therapeutic Effect of Atherosclerosis In Vivo

**Figure 6:**
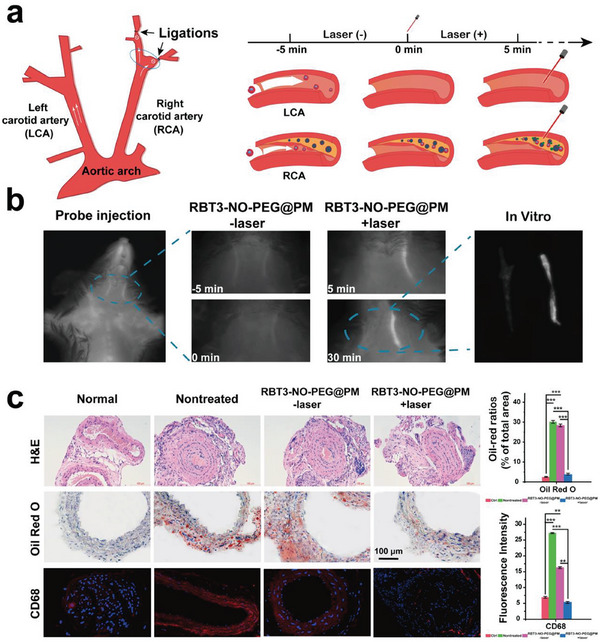
a) Diagrammatic sketch of carotid ligation surgery model. The right carotid artery (RCA) was ligated, and the left carotid artery (LCA) was regarded as the normal group. b) NIR‐II fluorescence imaging of artery sections after NO therapy. NIR‐II Fluorescence image of RBT3‐NO‐PEG@PM arriving at the plaque with blood circulation after injection through the tail vein of mice. After 5 min, the plaque was irradiated with an 808 nm laser at a fluence rate of 0.5 W cm^−2^. The injection concentration of RBT3‐NO‐PEG@PM is 5 mg kg^−1^ (Calculated as RBT3‐NO‐PEG). c) H&E, Oil Red O staining and CD68 imaging of artery sections after NO therapy (scale bar: 100 µm). The results are presented as mean ± SD (*n* = 6). Statistical significance was assessed applying one‐way ANOVA. ^*^ *p* < 0.05, ^**^ *p* < 0.01, ^***^ *p* < 0.001.

Next, we verified the anti‐atherosclerotic activity of the nanoparticles in model mice, and the results of hematoxylin‐eosin (HE) staining on the cross section of blood vessels confirmed the presence of atherosclerotic plaques in the vessels on the ligation side, which proved the successful construction of the model (**Figure** **6**). As shown in the Figure 6b and Figure S15 (Supporting Information), ≈5 min after the injection of RBT3‐NO‐PEG@PM into the tail vein of live mice, the nanoparticles were targeted and bound to the plaque sites in the blood circulation, and the fluorescence showed an obvious enhancement trend after laser irradiation, which was consistent with the in vitro spectral experiment and the in vitro arterial fluorescence imaging further confirmed the difference in the distribution of RBT3‐NO‐PEG@PM in the left and right neck blood vessels. Under laser stimulation, RBT3‐NO‐PEG@PM undergoes fluorescence enhancement, achieving specific localization of plaque sites and providing accurate guidance for treatment. On the contrary, the nano‐prodrug RBT3‐NO‐PEG without platelet membrane encapsulation cannot aggregate in the plaque area and has no obvious fluorescence signal under laser irradiation (Figure S16, Supporting Information). These results indicate that this platelet‐mimicking NO nano‐prodrug system RBT3‐NO‐PEG@PM can accurately target atherosclerotic plaque in vivo.

The ligation side oil red section also showed that the near infrared laser excitation achieved deep tissue penetration and full release of NO, thus reducing the lipid deposition in the lesion site, and further verified that the release of NO in mice was controllable (Figure S17, Supporting Information). As shown in Figure 6c, the lipid accumulation in the blood vessels of the RBT3‐NO‐PEG@PM laser irradiation group was significantly improved. After two weeks of RBT3‐NO‐PEG@PM injection assisted laser irradiation, the ligated lateral carotid artery in mice was removed and paraffin sections were taken for pathological analysis. The results of H&E sections in the non‐treated group showed signs of thrombosis, such as wall thickening, small cavity, and intima thickening, while the arrangement of the wall tissue was regular and thrombosis accumulation was significantly improved in the treatment group.

**Figure 7:**
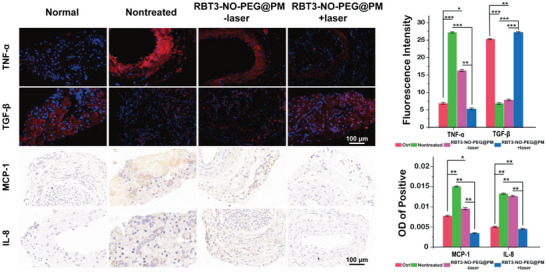
Immunofluorescence imaging of TNF‐α and TGF‐β and the imaging of MCP‐1 and IL‐8 in vascular slices after NO therapy (scale bar: 100 µm). The injection concentration of RBT3‐NO‐PEG@PM is 5 mg kg^−1^ (Calculated as RBT3‐NO‐PEG). The results are presented as mean ± SD (*n* = 6). Statistical significance was assessed applying one‐way ANOVA. ^*^ *p* < 0.05, ^**^ *p* < 0.01, ^***^ *p* < 0.001.

In addition, Macrophage marker CD68 immunofluorescence staining was used to confirm the decrease of foam cells and the improvement of lipid accumulation in the RBT3‐NO‐PEG@PM laser treatment group.^[^ ^26^ ^]^ The development of atherosclerosis is associated with chronic inflammation, so the improvement of lipid accumulation may also help reduce inflammation of the plaque area. As shown in **Figure** **7**, compared with the non‐treated group, the treatment group was much less TNF‐α, TGF‐β rise obviously,^[^ ^27^ ^]^ heralding the inflammation improved. At the same time, the expressions of chemokines MCP‐1 and IL‐8 were also significantly reduced in the treatment group,^[^ ^28^ ^]^ suggesting that NO therapy promotes endothelial cell proliferation and migration, improve the environment of inflammation. RBT3‐NO‐PEG@PM photocontrolled release NO treatment can transform the atherosclerotic plaque circumstance from pro‐inflammatory to anti‐inflammatory, thus easing the progression of atherosclerotic plaques. In addition, for a drug administered through intravenous injection, the distribution, metabolism, and safety are crucial. We conducted pharmacokinetic analysis on ApoE^−/−^ mice (Figure S18, Supporting Information). Compared with free RBT3‐NO (t_1/2_ = 0.88 h), the cyclic half‐life of RBT3‐NO‐PEG (t_1/2_ = 4.12 h) is approximately five times that of free RBT3‐NO, which will be conducive to the accumulation and treatment of RBT3‐NO‐PEG@PM in atherosclerotic plaque. The tissue distribution was shown in the Figure S19 (Supporting Information). In addition to reaching the plaque area on the right carotid artery, nanoparticles are also distributed in the main metabolic organs, liver, and kidney.

**Figure 8:**
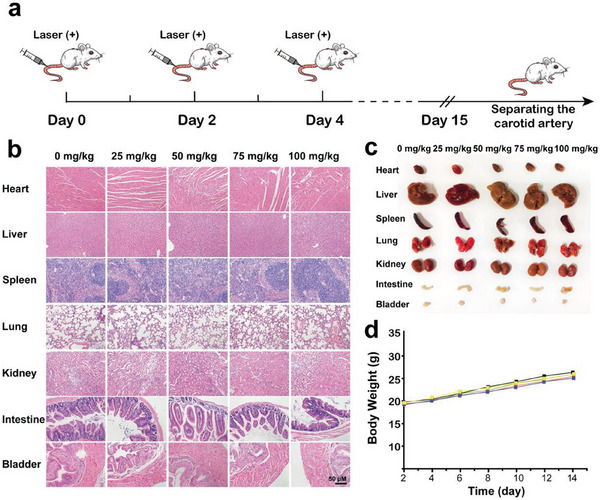
a) Treatment schedule of atherosclerotic plaques in mice. RBT3‐NO‐PEG@PM with a concentration of 5 mg kg^−1^ (Calculated as RBT3‐NO‐PEG) was injected every other day from day 0, and laser irradiation was performed half an hour after injection. b) H&E staining of sections from various organs after different therapies (scale bar: 50 µm). c) Photos of heart, liver, spleen, lung, kidney, intestine, and bladder after different treatments. d) Mice body weights after different treatments during 14 d (mean ± SD, *n* = 6).

In conclusion, the treatment of controlled release of NO by laser irradiation can reduce the accumulation of lipids at the spot of atherosclerotic plaques, improve the inflammatory response at the lesion site, promote the transformation of the plaque environment into an anti‐inflammatory environment, and thus slow the progression of plaques. At the same time, in order to evaluate whether RBT3‐NO‐PEG@PM has side effects on the body, histological analysis was conducted on mice treated with RBT3‐NO‐PEG@PM. HE staining analysis and morphological imaging of major organs in the whole body showed that there were no obvious injuries in all treatment groups, and the body weight of mice in each group had no significant change during treatment (**Figure** **8**). In addition, to further evaluate the biosafety of this platelet‐mimicking NO nano‐prodrug system, we conducted whole blood cell counts on mice treated with RBT3‐NO‐PEG@PM (Figure S20, Supporting Information). The whole blood cell count data showed that the levels of red blood cells, white blood cells, platelets, and hemoglobin in the treatment groups with different dosages were within the normal range. The results showed that RBT3‐NO‐PEG@PM nanoparticles had good biocompatibility and low biotoxicity even after continuous administration.

## 3 Conclusion

In conclusion, this study has developed an activatable platelet‐mimicking NO nano‐prodrug system RBT3‐NO‐PEG@PM for the diagnosis and treatment of atherosclerosis. The nano‐prodrug system can be effectively used to accurately locate atherosclerotic plaques through platelet membrane wrapping and control the release of NO through 808 nm laser irradiation. Its emission window reaches the NIR‐II area, opening an effective way for high‐resolution fluorescence imaging of deep tissue. The in vivo results showed that the NO nano‐prodrug system could reduce the accumulation of lipid in atherosclerotic plaques, improve the inflammatory reaction in the lesion, and promote endothelial cell migration. Therefore, we assume that our current research may afford a feasible way for the early diagnosis and treatment of clinical atherosclerosis.

## 4 Experimental Section

### Synthesis of RBT‐NH and RBT‐NO

See supporting literature for the synthesis methods and characterization data of all compounds.^[^ ^29^ ^]^

*General Procedure for the Synthesis of RBT‐NH*: A mixture of compound RBT‐Br, amine and cesium carbonate were added to a three‐necked flask containing 5 mL toluene. Then added palladium catalyst and ligand to the reaction liquid, stirred at room temperature for 5 min, and then refluxed for 5 h under nitrogen. After cooling to room temperature, added ice water to quench the reaction and extracted the organic layer with dichloromethane. The combined organic extracts were dried with anhydrous sodium sulfate, filtered, and evaporated. The crude product was further purified via silica column chromatography to afford compound RBT‐NH.

*General Procedure for the Synthesis of RBT‐NO*: A mixture of compound RBT‐NH and sodium nitrite were added to a single ended flask containing 6 mL tetrahydrofuran/methanol/water (3:2:1, v/v/v). Then acetic acid was added dropwise to the reaction liquid and stirred under 0 °C in darkness for 30 min. Then added water to quench the reaction and extracted the organic layer with dichloromethane. The combined organic extracts were dried with anhydrous sodium sulfate, filtered, and evaporated. The crude product was further purified via silica column chromatography to afford compound RBT‐NO.

### Preparation of RBT3‐NO‐PEG@PM

DSPE‐mPEG_5k_ (10 mg) was added in 1 mL normal saline and stirred at room temperature until completely dispersed with 1 mg RBT3‐NO which was weighed and completely dissolved in 10 µL DMSO, and then slowly dropped into it. After stirring overnight, it was filtered through filter membrane to obtain RBT3‐NO‐PEG. Whole blood of ApoE^−/−^ mice was collected by venous sinus method and placed in an anticoagulant centrifuge tube. The blood was centrifuged at 800 rpm for 10 min after standing for 10 min and the supernatant was placed in a clean centrifuge tube and centrifuged at 3500 rpm for 10 min. After discarding the supernatant, the bottom of the tube was precipitated and the hypotonic saline with four degrees of precooling was added to extract the platelet membrane vesicles by hypotonic dilation and centrifugation for several times to remove the red blood cells. The platelet membrane vesicles extracted by hypoosmotic burst method were mixed with RBT3‐NO‐PEG at a weight ratio of 1:1 and the mixture was shaken in a water bath at 37 °C to make the contents uniformly dispersed and slightly transparent. The membrane fusion was carried out by ultrasonic extrusion method, and the platelet membrane was coated on RBT3‐NO‐PEG to obtain the final RBT3‐NO‐PEG@PM.

### Validation of Intracellular NO Release

The cytotoxicity of RBT3‐NO‐PEG@PM was first verified before cell experiments. RAW and HUVECs cells were seeded in 96‐well plates for 24 h, and then RBT3‐NO‐PEG@PM of 0–25 µg mL^−1^ (Calculated as RBT3‐NO‐PEG) were loaded into cells for 24 and 48 h. Finally, 10 µL MTT was added to each well and incubated for 4 h, then 150 µL DMSO was substituted. After shaking for 10 min, absorbance values of different groups at 490 nm were collected to obtain the cytotoxic activity of RBT3‐NO‐PEG@PM. In the same way, the MTT assay of RBT3‐NO and RBT3 co‐cultured for 24 h in RAW cells and HUVECs cells was performed.

Next, the ability of RBT3‐NO‐PEG@PM to release NO was verified at the cellular level by using a NO indicator DAF‐FM‐DA. RAW and HUVECs cells (5 × 10^4^ mL^−1^) were inoculated in confocal culture dishes for 24 h to make the cells stick to the wall and 15 µg mL^−1^ RBT3‐NO‐PEG@PM was added and incubated for 3 h. After that, the cells were washed three times with PBS, 5 mM DAF‐FM‐DA was added, and imaging was performed under confocal fluorescence microscopy.

RAW and HUVECs cells (5 × 10^4^ mL^−1^) were inoculated in confocal culture dishes for 24 h. RBT3‐NO‐PEG@PM were added in the dish with the final concentration of 15 µg mL^−1^ and incubated in the dark for 3 h. After that, the cells were washed three times with PBS. The NO indicator DAF‐FM‐DA was dissolved in DMSO with the final concentration of 20 µm. Then, the two were co‐incubated in the same dish for another 0.5 h in the dark and the cells were ready for the irritation of NIR laser light (808 nm, 0.5 W cm^−2^). Finally, the collected cells were washed and centrifuged with cold PBS buffer, and cells were resuspended with 500 µL of cold PBS buffer and signals were collected using flow cytometry.

### Oil Red O Staining of Foam Cells

The ability of RBT3‐NO‐PEG@PM to improve lipid accumulation was verified by oil red O staining of foam cells. The foam cells were transformed from primary peritoneal macrophages incubated with oxidized low‐density lipoprotein (ox‐LDL). Ox‐LDL of 50 µg mL^−1^ was incubated with macrophages for >48 h to obtain lipid‐filled foam cells. 15 µg mL^−1^ RBT3‐NO‐PEG@PM (Calculated as RBT3‐NO‐PEG) was added into the foam cells, and the treatment group was irradiated with laser light (808 nm, 0.5 W cm^−2^). After that, the cells of different groups were stained with oil red O, and the oil red O working solution was stained at room temperature for 30 min, and the staining result was recorded by microscope.

### Cell Scratch Assay

The ability of RBT3‐NO‐PEG@PM to promote endothelial cell migration was verified by scratch experiments. The cells of HUVECs were inoculated into the six‐well plate so that the cells uniformly grew to just cover the entire bottom of the hole plate, and then made a vertical scratch on the bottom of the hole plate with the tip of pipetting head. After the floating cells were washed off with PBS, serum‐free medium was replaced and 15 µg mL^−1^ RBT3‐NO‐PEG@PM (Calculated as RBT3‐NO‐PEG) was added for co‐incubated in the experimental group and the cell migration was recorded under microscope.

### In Vivo Imaging of Atherosclerotic Mice

ApoE knockout homozygous mice (ApoE^−/−^) were used to induce atherosclerosis by ligation of the right carotid artery combined with high‐fat diet. After feeding a high‐fat diet for 1 month, the mice were injected intravenously with RBT3‐NO‐PEG@PM (5 mg kg^−1^, calculated as RBT3‐NO‐PEG), and fluorescence imaging was performed at different points of laser irradiation (808 nm, 0.5 W cm^−2^) for 0, 10, and 30 min. The injection method was through the tail vein of mice, and the detailed operating steps are as follows: First, grab and fix the mice and disinfect their tails with alcohol. Then, insert the needle of the syringe with an angle of 30–45 degree angle upward from the skin into the tail vein. After insertion, the needle gently swings left and right, and then gently aspirates. If there was no blood return, the drug could be slowly injected subcutaneously. After injection, remove the needle and press the injection site with a sterile cotton swab for a moment to prevent drug leakage.

### Evaluation of RBT3‐NO‐PEG@PM in Improving Atherosclerosis In Vivo

Mice were randomly divided into three groups, which were injected with 1) normal saline 2) 5 mg kg^−1^ RBT3‐NO‐PEG@PM (Calculated as RBT3‐NO‐PEG) and 3) 5 mg kg^−1^ RBT3‐NO‐PEG@PM with irradiated by laser through caudal vein, respectively. RBT3‐NO‐PEG@PM was injected every other day from day zero, and 30 min after injection, the carotid artery ligation site of mice was irradiated by laser for 30 min (808 nm, 0.5 W cm^−2^). During the treatment, the weight changes of mice were recorded regularly, and the treatment lasted for 15 days. Fifteen days later, the mice were euthanized and their LCA was removed for pathological analysis.

The decrease of macrophage‐derived foam cells might be the cause of the decrease of lipid accumulation in vivo. Therefore, immunofluorescence staining of macrophage marker CD68 could verify the improvement effect of RBT3‐NO‐PEG@PM on lipid accumulation. The effect of RBT3‐NO‐PEG@PM on improving atherosclerosis in vivo could be further evaluated by oil red O staining of blood vessels at focal sites. And for several inflammatory cytokines, including proinflammatory tumor necrosis factor‐α (TNF‐α) and anti‐inflammatory transforming growth factor‐β (TGF‐β) expression level of immune fluorescence imaging in the evaluation of the overall state of inflammation in mice. Monocyte chemokines 1 (MCP‐1) and interleukin‐8 (IL‐8) are chemokines that perform an important role in inflammation and their upregulation could scale up the adhesion of vascular endothelial cells and blood cells, affect vascular permeability and damage vascular intima. Evaluating the expression of these two chemokines can also assess the progression of atherosclerosis in mice.

## Conflict of Interest

The authors declare no conflict of interest.

## Supporting information

Supporting Information

## Data Availability

The data that support the findings of this study are available in the supplementary material of this article.;
