# Supplementary material for: Near Infrared Light‐Activatable Platelet‐Mimicking NIR‐II NO Nano‐Prodrug for Precise Atherosclerosis Theranostics
Source: Adv Sci (Weinh). 2023 Nov 30;11(3):2304994. doi: 10.1002/advs.202304994 (PMC10797437; doi:10.1002/advs.202304994)
Supplement: Supplementary file 1 — Supporting Information [file ADVS-11-2304994-s001.pdf]

ADVANCED SCIENCE
Open Access

Supporting Information

for *Adv. Sci.*, DOI 10.1002/advs.202304994

Near Infrared Light-Activatable Platelet-Mimicking NIR-II NO Nano-Prodrug for Precise Atherosclerosis Theranostics

Yun Chai, Lina Shangguan, Hui Yu, Ye Sun, Xiaoyan Huang, Yanyan Zhu, Hai-Yan Wang and Yi Liu**

Supporting information

Near Infrared Light-Activatable Platelet-Mimicking NIR-II NO Nano-Prodrug for Precise Atherosclerosis Theranostics

Yun Chai[a], *Lina Shangguan*[a], *Hui Yu*[a], *Ye Sun*[a], *Xiaoyan Huang*[a], *Yanyan Zhu*[a], *Hai-Yan Wang*[b]*, *Yi Liu*[a]*

Table of Contents

1. Materials and Instrumentals..2

2. Phototriggered and Control Release Assay 2

3. In vitro NO Detection Assay 2

4. Cell Culture 3

5. Cytotoxicity Assay..3

6. Intracellular NO Release Assay...3

7. Animal Model..4

8. Pharmacokinetic Experiments...5

9. Chemical synthesis of RBT-NH and RBT-NO 5

10. Supplementary Figures 11

11. Structural Characterization...23

1. Materials and Instrumentals

The chemical reagents used in the synthesis of these compounds were purchased from China Reagent Network. The ^{1}H-NMR and ^{13}C-NMR data were obtained either through Bruker 300 MHz or Bruker 400 MHz. Mass spectroscopy data was collected on Waters Q-TOF MicroTM. High performance liquid chromatography (HPLC) data were obtained via Thermo Scientific dionex ultimate 3000. UV-Vis and fluorescence spectra were recorded on a Shimadzu UV-Vis spectrophotometer, UV-3600 Plus and Edinburgh Instruments Spectrofluorometer, FLS-1000, respectively. Fluorescence imaging of cells were carried out by a confocal laser canning microscopy (CLSM, LSM800, Zeiss, Germany). Fluorescence imaging of mice was performed on an small animal optical in vivo imaging system (Dali-IGS 600, China).

2. Phototriggered and Control Release Assay

Dissolve RBT-NO (10 μM) and RBT3-NO-PEG@PM (10 μM) in dichloromethane and aqueous solution respectively. Then, exposed to NIR laser (808 nm, 0.5 W cm^{-2}), record UV absorption spectrum and fluorescence emission spectrum respectively (λ_{ex} = 808 nm). The same procedure shall be applied to the controlled release measurement, and the NO donor shall be conducted alternately between the laser on and the laser off environment for 10 minutes.

3. In vitro NO Detection Assay

RBT-NO (10 μM) were dissolved in 3 mL of analytically pure methanol and purified water (v/v, 1:1), RhBs (10 μM) were added to the quartz cuvette in liquid form that is pre-dissolved in DMSO. Then, the two compounds were exposed to light

irritation (808 nm, 0.5 W cm^{-2}) for 70 min while the fluorescent turn-on was recorded with fluorescent spectra (λ_{ex} = 545 nm).

4. Cell Culture

Cells were cultured in DMEM with 10% FBS. Generally, cells were maintained under a humidified atmosphere containing 5% CO_2 at 37 °C. Experiments were performed either in 96-well plates and confocal dish.

5. Cytotoxicity Assay

RAW and HUVECs cells were seeded in 96-well plates for 24 h at 37 °C. Further, the media was removed from the plate and added fresh media 190 μL. Meanwhile, RBT3-NO-PEG@PM diluted by media and added to per well 10 μL with final concentration of 25 μg mL^{-1}, 20 μg mL^{-1}, 15 μg mL^{-1}, 10 μg mL^{-1} and 5 μg mL^{-1}. 96-well plate was incubated for 24 h and 48 h carefully wrapped with silver paper. A solution of MTT was added to each well 10 μL before incubated for another 4 h. the media was removed from the 96-well and replaced with 100 μL DMSO. Cytotoxicity and proliferation were acquired by comparing the absorbance of each well with the absorbance of the control wells at 490 nm. The cytotoxic experiment of RBT3-NO-PEG@PM was completed as described above. In the same way, the MTT assay of RBT3-NO and RBT3 co-cultured for 24 hours in RAW cells and HUVECs cells was performed.

6. Intracellular NO Release Assay

RAW and HUVECs cells ($5x10^4$ mL^{-1}) were inoculated in confocal culture dishes for 24 h. The media in the dish were replaced with fresh one, meanwhile,

RBT3-NO-PEG@PM were added in the dish with the final concentration of 15 μg mL^{-1} and incubated in the dark for 3 h. The NO indicator DAF-FM-DA was dissolved in DMSO with the final concentration of 20 μM. Then, the two were co-incubated in the same dish for another 0.5 h in the dark. After that, the dish was rinsed with PBS for three times before added 100 μL polyoxymethylene for cells fixation. Then, the cells were ready for the irritation of NIR laser light (808 nm, 0.5 W cm^{-2}) and the detection of intracellular NO release, which was acquired with confocal laser canning microscopy (CLSM, LSM800, Zeiss, Germany) equipped with a 63×oil objective lens. The green fluorescence (FITC channel) was excited with 492 nm. For the image acquisition and statistical analysis, the Zen 2008 software was used.

RAW and HUVECs cells ($5x10^4$ mL^{-1}) were inoculated in confocal culture dishes for 24 h. The media in the dish were replaced with fresh one, meanwhile, RBT3-NO-PEG@PM were added in the dish with the final concentration of 15 μg mL^{-1} and incubated in the dark for 3 h. After that, the cells were washed three times with PBS. The NO indicator DAF-FM-DA was dissolved in DMSO with the final concentration of 20 μM. Then, the two were co-incubated in the same dish for another 0.5 h in the dark and the cells were ready for the irritation of NIR laser light (808 nm, 0.5 W cm^{-2}). After that, the dish was rinsed with PBS for three times before added pancreatin. Finally, the collected cells were washed and centrifuged with cold PBS buffer, and cells were resuspended with 500 μL of cold PBS buffer and signals were collected using flow cytometry.

7. Animal Model

ApoE knockout homozygous mice ($ApoE^{-/-}$) were purchased from Qinglongshan, and at the age of 5-6 weeks the atherosclerosis model was establish by ligation of the

right carotid artery combined with high-fat diet. After feeding a high-fat diet for one month, the mice were injected intravenously with RBT3-NO-PEG@PM (5 mg kg^{-1}), and fluorescence imaging was performed at different points of laser irradiation (808 nm, 0.5 W cm^{-2}) for 0 min, 10 min, and 30 min. All the animal experiments were approved by the ethics committee of China Pharmaceutical University and conducted with the “guide for the care and use of laboratory animals” of the institute of laboratory animal resources.

8. Pharmacokinetic Experiments

ApoE knockout homozygous mice ($ApoE^{-/-}$) were used to induce atherosclerosis by ligation of the right carotid artery combined with high-fat diet. After feeding a high-fat diet for one month, the mice were divided into two groups, with 3 mice in each group, and injected RBT3-NO (0.8 mg kg^{-1}) and RBT3-NO-PEG@PM (5 mg kg^{-1}, calculated as RBT3-NO-PEG) into the tail vein respectively. 30 μL blood samples were taken from the tail of mice at different time points, stored in a centrifuge tube coated with heparin sodium solution, and 0.3% triton X-100 was added for ultrasound for 3 minutes. Then add 300 μL DMSO solution and centrifuge at 10000 rpm for 15 minutes. Finally, the supernatant was taken and its drug concentration was determined by HPLC.

9. Chemical synthesis of RBT-NH and RBT-NO

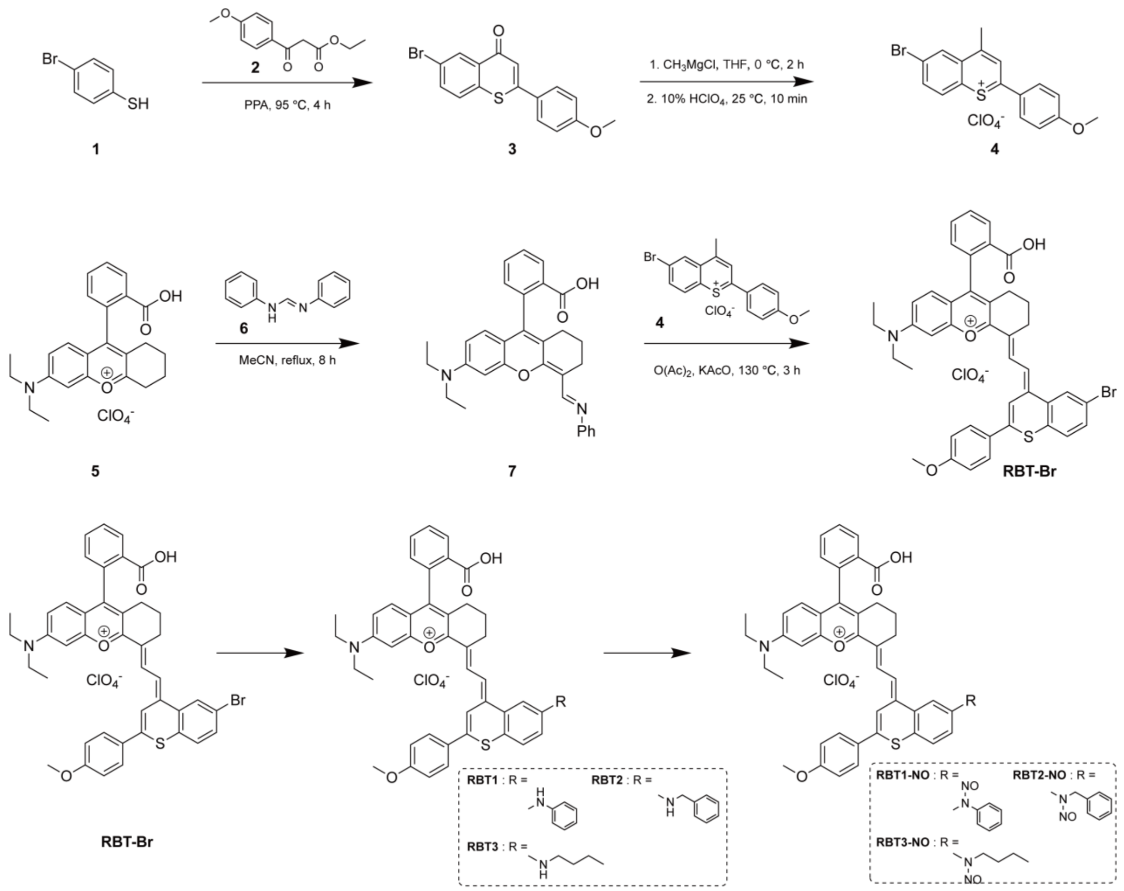

Scheme S1. Synthetic route for RBT-NH and RBT-NO.

Synthesis of compound 3: A mixture of polyphosphoric acid (PPA, 22 g), compound 1 (2.0 g, 10.6 mmol), and compound 2 (2.6 g, 11.66 mmol) was stirred at 95 ℃ for 4 h. After cooling to room temperature, added ice water to quench the reaction and extracted the organic layer with DCM (100 mL × 3). The combined organic extracts were dried with Na_2SO_4, filtered, and evaporated. The crude product was further purified via silica column chromatography in PE/DCM (1:1 - 0:1, v/v) to afford compound 3 (yield: 1.3 g, 35%). ^{1}H NMR (300 MHz, $CDCl_3$) δ 8.69 (s, 1H), 7.73 (d, *J* = 9.0 Hz, 1H), 7.66 (d, *J* = 9.0 Hz, 2H), 7.54 (d, *J* = 9.0 Hz, 1H), 7.24 (s, 1H), 7.03 (d, *J* = 9.0 Hz, 2H), 3.90 (s, 3H).

Synthesis of compound 4: Dissolve compound 3 (200 mg, 0.59 mmol) in anhydrous THF (5 mL) under nitrogen in a dry flask. Add 3.0 M CH_3MgCl (0.6 mL,

1.80 mmol) dropwise to the solution at 0 °C and stir at room temperature for 2 h. Drop the reaction liquid into a 10% $HClO_4$ aqueous solution (10 mL), stir for 5 minutes, and then filter to separate the orange red solid. The orange red solid was washed 3 times with diethyl ether (15 mL) and dried to obtain compound 4 (yield: 220 mg, 86%). ^{1}H NMR (300 MHz, CD_3CN) δ 9.02 (s, 1H), 9.01 (s, 1H), 8.53 (d, *J* = 9.0 Hz, 1H), 8.41 (d, *J* = 9.0 Hz, 1H), 8.35 (d, *J* = 9.0 Hz, 2H), 7.43 (d, *J* = 9.0 Hz, 2H), 4.13 (s, 3H), 3.28 (s, 3H). HRMS (ESI): calcd for, $C_{17}H_{14}BrOS^+$ 344.9943 $[M]^+$; found 344.9936.

Synthesis of compound 7: A mixture of compound 5 (1.0 g, 2.1 mmol) and compound 6 (618.4 mg, 3.15 mmol) was added to a flask containing 5 mL MeCN, stirred at room temperature for 5 minutes, and then refluxed for 8 h under N_2. After cooling to room temperature, the reaction liquid was evaporated to obtain the crude product. The crude product was washed 3 times with diethyl ether (15 mL) and dried to obtain compound 7 (yield: 0.9 g, 90%). ^{1}H NMR (300 MHz, $CDCl_3$) δ 7.70 - 7.50 (m, 3H), 7.40 - 7.30 (m, 4H), 7.26 (s, 1H), 7.22 - 7.15 (m, 2H), 7.10 (d, *J* = 9.0 Hz, 1H), 6.78 (s, 1H), 6.72 (d, *J* = 6.0 Hz, 1H), 3.54 - 3.47 (m, 6H), 2.60 - 2.55 (m, 1H), 2.17 - 2.15 (m, 1H), 1.28 - 1.21 (m, 8H).

Synthesis of compound RBT-Br: A mixture of compound 7 (237 mg, 0.50 mmol), compound 4 (200 mg, 0.45 mmol) and KOAc (49 mg, 0.50 mmol) was added to a three-necked flask containing 5 mL of $O(Ac)_2$, stirred at room temperature for 5 minutes, and then heated for 3 h under N_2 at 130 °C. After cooling to room temperature, the reaction liquid was evaporated to obtain the crude product. The crude product was further purified via silica column chromatography in DCM/MeOH (200:1 - 15:1, v/v) to afford compound RBT-Br (yield: 180 mg, 48%). ^{1}H NMR (300 MHz, $CDCl_3$) δ 8.10 (d, *J* = 9.0 Hz, 1H), 7.90 (s, 1H), 7.72 - 7.54 (m, 5H), 7.46 (s, 1H), 7.37

(d, J = 9.0 Hz, 1H), 7.22 - 7.16 (m, 2H), 6.97 (d, J = 6.0 Hz, 2H), 6.77 (d, J = 12.0 Hz, 1H), 6.68 (d, J = 9.0 Hz, 1H), 6.52 (d, J = 9.0 Hz, 2H), 3.87 (s, 3H), 3.47 - 3.45 (m, 4H), 2.75 - 2.68 (m, 2H), 1.83 - 1.76 (m, 2H), 1.27 - 1.22 (m, 8H). ^{13}C NMR (75 MHz, DMSO-d_6) δ 167.15, 162.79, 161.45, 133.71, 132.94, 132.11, 131.67, 130.58, 130.11, 129.53, 129.42, 128.77, 128.35, 122.29, 117.86, 117.35, 114.88, 96.52, 55.86, 55.40, 45.66, 31.62, 30.29, 29.48, 26.19, 25.04, 22.58, 20.98, 14.44, 12.98. HRMS (ESI): calcd for, $C_{42}H_{37}BrNO_4S^+$ 730.1621 $[M]^+$; found 730.1611.

Synthesis of compound RBT1: A mixture of compound RBT-Br (100 mg, 0.12 mmol), aniline (16.85 mg, 0.18 mmol) and Cs_2CO_3 (118.6 mg, 0.36 mmol) was added to a three-necked flask containing 5 mL toluene. Then added $Pd_2(dba)_3$ (11.02 mg, 0.012 mmol) and Xphos (17.25 mg, 0.036 mmol) to the reaction liquid, stirred at room temperature for 5 minutes, and then refluxed for 5 h under N_2. After cooling to room temperature, added ice water to quench the reaction and extracted the organic layer with DCM (30 mL × 3). The combined organic extracts were dried with Na_2SO_4, filtered, and evaporated. The crude product was further purified via silica column chromatography in DCM/MeOH (200:1 - 15:1, v/v) to afford compound RBT1 (yield: 45 mg, 44%). ^{1}H NMR (300 MHz, $CDCl_3$) δ 8.01 (d, J = 9.0 Hz, 1H), 7.69 - 7.56 (m, 6H), 7.45 (s, 1H), 7.34 - 7.31 (m, 2H), 7.26 - 7.20 (m, 2H), 7.14 - 7.08 (m, 3H), 6.97 (d, J = 6.0 Hz, 3H), 6.79 (d, J = 12.0 Hz, 1H), 6.56 (d, J = 9.0 Hz, 1H), 6.43 - 6.38 (m, 2H), 6.02 (s, 1H), 3.88 (s, 3H), 3.41 - 3.39 (m, 4H), 2.80 - 2.59 (m, 2H), 2.12 - 2.03 (m, 2H), 1.66 - 1.63 (m, 2H), 1.24 - 1.20 (m, 8H). HRMS (ESI): calcd for, $C_{48}H_{43}N_2O_4S^+$ 743.2938 $[M]^+$; found 743.2935.

Synthesis of compound RBT2: A mixture of compound RBT-Br (100 mg, 0.12 mmol), benzylamine (19.4 mg, 0.18 mmol) and Cs_2CO_3 (118.6 mg, 0.36 mmol) was added to a three-necked flask containing 5 mL toluene. Then added $Pd(OAc)_2$ (2.8 mg,

0.012 mmol) and BrettPhos (19.4 mg, 0.036 mmol) to the reaction liquid, stirred at room temperature for 5 minutes, and then refluxed for 5 h under N_2. After cooling to room temperature, added ice water to quench the reaction and extracted the organic layer with DCM (30 mL × 3). The combined organic extracts were dried with Na_2SO_4, filtered, and evaporated. The crude product was further purified via silica column chromatography in DCM/MeOH (200:1 - 15:1, v/v) to afford compound RBT2 (yield: 30 mg, 29%). ^{1}H NMR (300 MHz, $CDCl_3$) δ 8.07 (d, J = 9.0 Hz, 1H), 7.72 - 7.54 (m, 6H), 7.46 - 7.41 (m, 3H), 7.37 - 7.32 (m, 2H), 7.27 - 7.24 (m, 1H), 7.19 - 7.13 (m, 2H), 7.02 (s, 1H), 6.95 (d, J = 9.0 Hz, 2H), 6.75 (d, J = 9.0 Hz, 1H), 6.63 (d, J = 9.0 Hz, 2H), 6.45 - 6.42 (m, 2H), 4.41 (s, 2H), 3.86 (s, 3H), 3.45 - 3.38 (m, 4H), 2.63 - 2.53 (m, 2H), 2.20 - 2.14 (m, 2H), 1.80 - 1.71 (m, 2H), 1.31 - 1.20 (m, 8H). HRMS (ESI): calcd for, $C_{49}H_{45}N_2O_4S^+$ 757.3095 $[M]^+$; found 757.3090.

Synthesis of compound RBT3: A mixture of compound RBT-Br (100 mg, 0.12 mmol), n-butylamine (13.2 mg, 0.18 mmol) and Cs_2CO_3 (118.6 mg, 0.36 mmol) was added to a three-necked flask containing 5 mL toluene. Then added $Pd(OAc)_2$ (2.8 mg, 0.012 mmol) and BrettPhos (19.4 mg, 0.036 mmol) to the reaction liquid, stirred at room temperature for 5 minutes, and then refluxed for 5 h under N_2. After cooling to room temperature, added ice water to quench the reaction and extracted the organic layer with DCM (30 mL × 3). The combined organic extracts were dried with Na_2SO_4, filtered, and evaporated. The crude product was further purified via silica column chromatography in DCM/MeOH (200:1 - 15:1, v/v) to afford compound RBT3 (yield: 22 mg, 22%). ^{1}H NMR (300 MHz, $CDCl_3$) δ 8.17 (d, J = 9.0 Hz, 1H), 7.93 (d, J = 12.0 Hz, 1H), 7.67 - 7.55 (m, 5H), 7.22 - 7.11 (m, 5H), 6.95 (d, J = 9.0 Hz, 2H), 6.85 - 6.72 (m, 3H), 6.56 - 6.49 (m, 2H), 3.86 (s, 3H), 3.48 - 3.43 (m, 4H), 2.71 - 2.68 (m, 2H), 2.29 - 2.24 (m, 2H), 1.83 - 1.78 (m, 2H), 1.69 - 1.64 (m, 2H), 1.50 - 1.43 (m, 2H),

1.31 - 1.23 (m, 11H). HRMS (ESI): calcd for, $C_{46}H_{47}N_2O_4S^+$ 723.3251 $[M]^+$; found 723.3249.

Synthesis of compound RBT1-NO: A mixture of compound RBT1 (35 mg, 0.042 mmol) and $NaNO_2$ (13.8 mg, 0.20 mmol) was added to a single ended flask containing 6 mL THF/MeOH/H_2O (3:2:1, v/v/v). Then AcOH (24 mg, 0.40 mmol) was added dropwise to the reaction liquid, and stirred under 0 °C in darkness for 30 minutes. Then added water to quench the reaction and extracted the organic layer with DCM (10 mL × 3). The combined organic extracts were dried with Na_2SO_4, filtered, and evaporated. The crude product was further purified via silica column chromatography in DCM/MeOH (200:1 - 15:1, v/v) to afford compound RBT1-NO (yield: 8 mg, 22%). ^{1}H NMR (300 MHz, $CDCl_3$) δ 8.04 - 8.01 (m, 1H), 7.71 - 7.61 (m, 2H), 7.66 - 7.54 (m, 4H), 7.50 - 7.43 (m, 4H), 7.38 - 7.35 (m, 1H), 7.22 - 7.09 (m, 3H), 7.00 - 6.94 (m, 3H), 6.81 - 6.69 (m, 1H), 6.59 - 6.54 (m, 1H), 6.45 - 6.40 (m, 2H), 3.89 (s, 3H), 3.45 - 3.38 (m, 4H), 2.76 - 2.51 (m, 2H), 2.30 - 2.23 (m, 2H), 1.76 - 1.72 (m, 2H), 1.28 - 1.25 (m, 8H). HRMS (ESI): calcd for, $C_{48}H_{42}N_3O_5S^+$ 772.2840 $[M]^+$; found 772.4598.

Synthesis of compound RBT2-NO: A mixture of compound RBT2 (35 mg, 0.041 mmol) and $NaNO_2$ (13.8 mg, 0.20 mmol) was added to a single ended flask containing 6 mL THF/MeOH/H_2O (3:2:1, v/v/v). Then AcOH (24 mg, 0.40 mmol) was added dropwise to the reaction liquid, and stirred under 0 °C in darkness for 30 minutes. Then added water to quench the reaction and extracted the organic layer with DCM (10 mL × 3). The combined organic extracts were dried with Na_2SO_4, filtered, and evaporated. The crude product was further purified via silica column chromatography in DCM/MeOH (200:1 - 15:1, v/v) to afford compound RBT2-NO (yield: 5 mg, 14%). ^{1}H NMR (300 MHz, $CDCl_3$) δ 8.29 (s, 1H), 8.13 (d, J = 9.0 Hz,

1H), 7.73 (d, J = 9.0 Hz, 2H), 7.66 - 7.54 (m, 4H), 7.38 - 7.37 (m, 2H), 7.22 - 7.11 (m, 6H), 7.05 (d, J = 9.0 Hz, 2H), 6.86 - 6.75 (m, 2H), 6.64 - 6.56 (m, 2H), 4.81 - 4.79 (m, 3H), 4.42 (d, J = 9.0 Hz, 1H), 3.91 (s, 3H), 3.47 - 3.44 (m, 4H), 2.39 - 2.22 (m, 4H), 1.31 - 1.27 (m, 8H). HRMS (ESI): calcd for, $C_{49}H_{44}N_3O_5S^+$ 786.2996 $[M]^+$; found 786.2989.

Synthesis of compound RBT3-NO: A mixture of compound RBT3 (30 mg, 0.036 mmol) and $NaNO_2$ (12.4 mg, 0.18 mmol) was added to a single ended flask containing 6 mL THF/MeOH/H_2O (3:2:1, v/v/v). Then AcOH (21.6 mg, 0.36 mmol) was added dropwise to the reaction liquid, and stirred under 0 °C in darkness for 30 minutes. Then added water to quench the reaction and extracted the organic layer with DCM (10 mL × 3). The combined organic extracts were dried with Na_2SO_4, filtered, and evaporated. The crude product was further purified via silica column chromatography in DCM/MeOH (200:1 - 15:1, v/v) to afford compound RBT3-NO (yield: 5 mg, 16%). ^1H NMR (300 MHz, $CDCl_3$) δ 8.28 (s, 1H), 8.19 - 8.12 (m, 1H), 7.81 - 7.73 (m, 2H), 7.66 - 7.54 (m, 2H), 7.37 - 7.34 (m, 1H), 7.14 - 7.01 (m, 3H), 6.91 - 6.65 (m, 3H), 6.36 - 6.12 (m, 2H), 5.85 - 5.62 (m, 2H), 5.39 - 5.35 (m, 1H), 3.94 - 3.91 (m, 3H), 3.54 - 3.52 (m, 2H), 3.35 - 3.19 (m, 4H), 2.78 - 2.74 (m, 2H), 2.28 - 2.23 (m, 2H), 1.68 - 1.63 (m, 2H), 1.53 - 1.47 (m, 2H), 1.31 - 1.28 (m, 11H). HRMS (ESI): calcd for, $C_{46}H_{46}N_3O_5S^+$ 752.3153 $[M]^+$; found 752.3158.

10. Supplementary Figures

Table S1: Photophysical properties of RBT-NH.

compound	λ_{abs}(nm)	λ_{em}(nm)	Stokes shift/nm	ε (M^{-1} cm^{-1})	Φ(%)
RBT-Br	763	933	170	39300	0.063
RBT1	869	928	59	26000	0.071

RBT2	870	929	59	33600	0.09
RBT3	872	933	61	39700	0.106

[a] Photophysical properties in dichloromethane. [b] For determination of the fluorescence quantum effificiency, IR26 in dichloroethane (Φ = 0.05%) was used as a fluorescence standard.

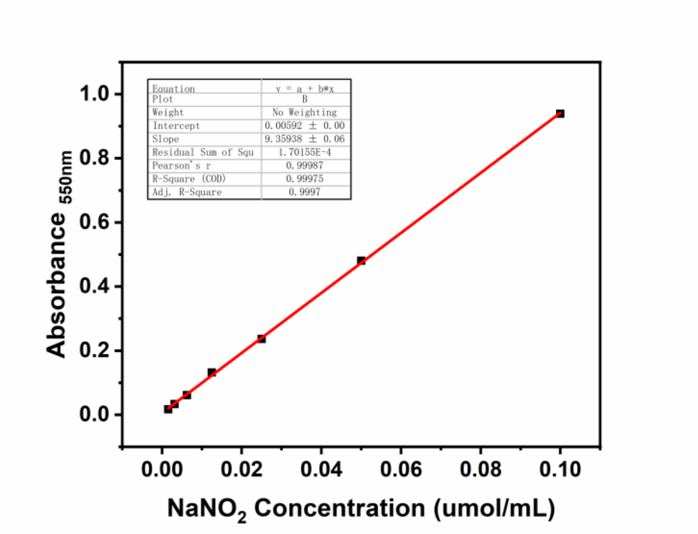

Figure S1. Working curve of Griess NO quantification methods.

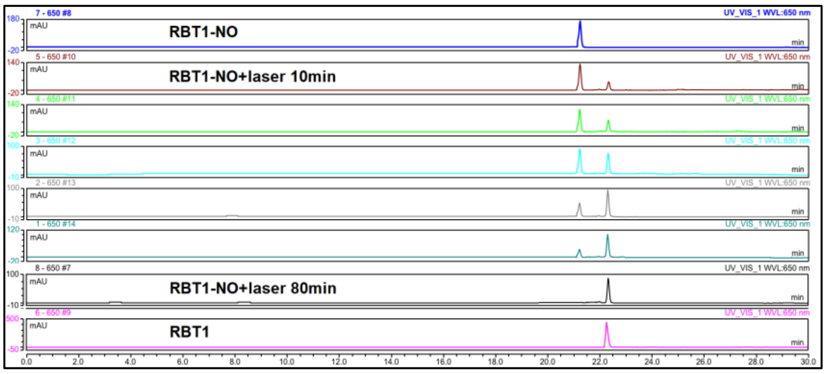

Figure S2. HPLC monitoring of the photolysis of RBT1-NO, while it was irritation with NIR light (808 nm, 0.5 W cm^{-2}, 80 min).

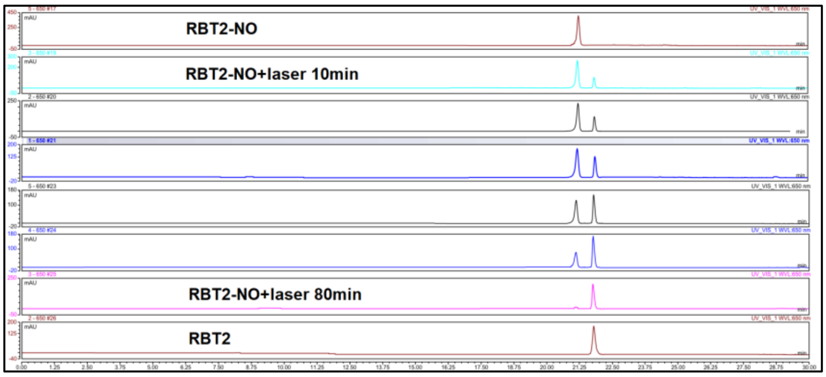

Figure S3. HPLC monitoring of the photolysis of RBT2-NO, while it was irritation with NIR light (808 nm, 0.5 W cm^{-2}, 80 min).

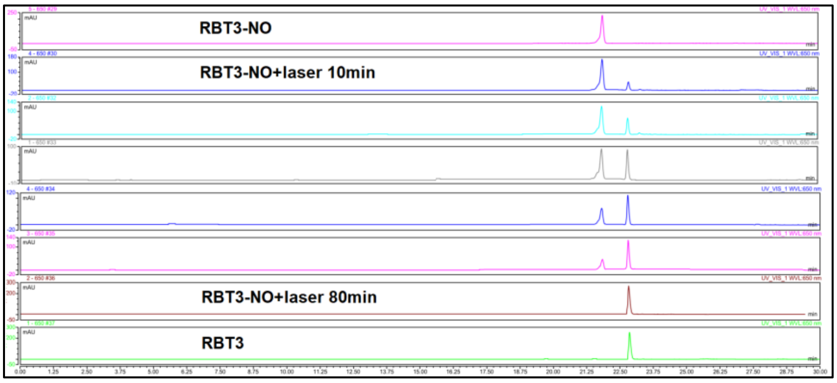

Figure S4. HPLC monitoring of the photolysis of RBT3-NO, while it was irritation with NIR light (808 nm, 0.5 W cm^{-2}, 80 min).

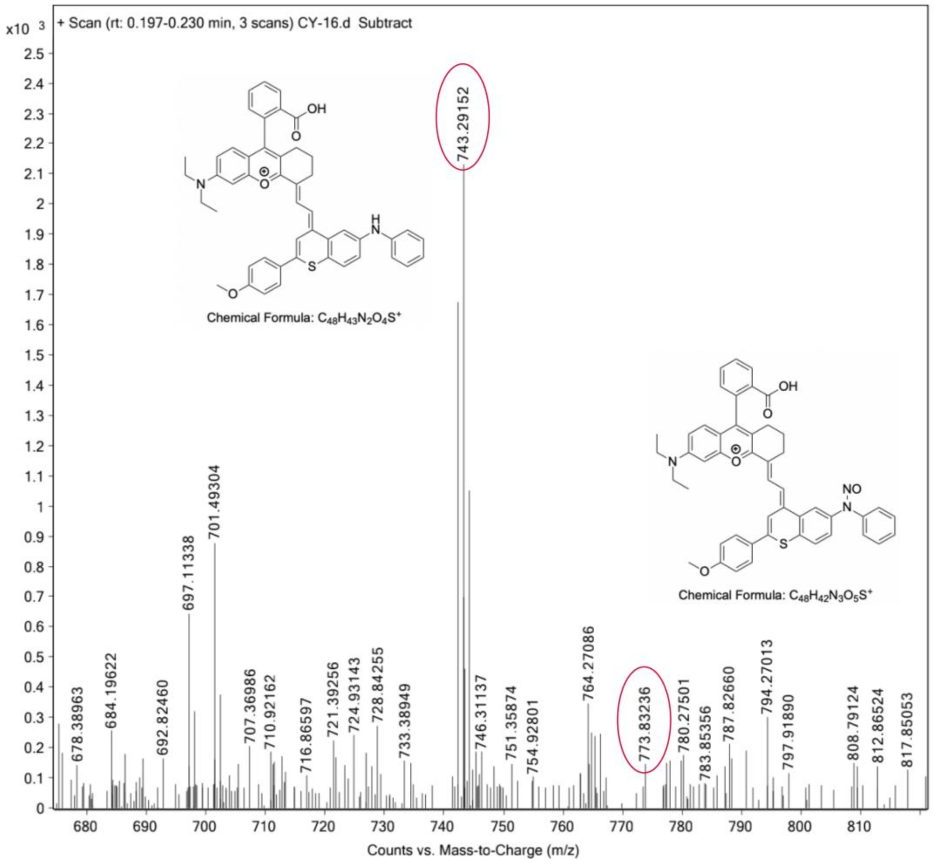

Figure S5. HRMS of HPLC monitored photo-release of RBT1-NO.

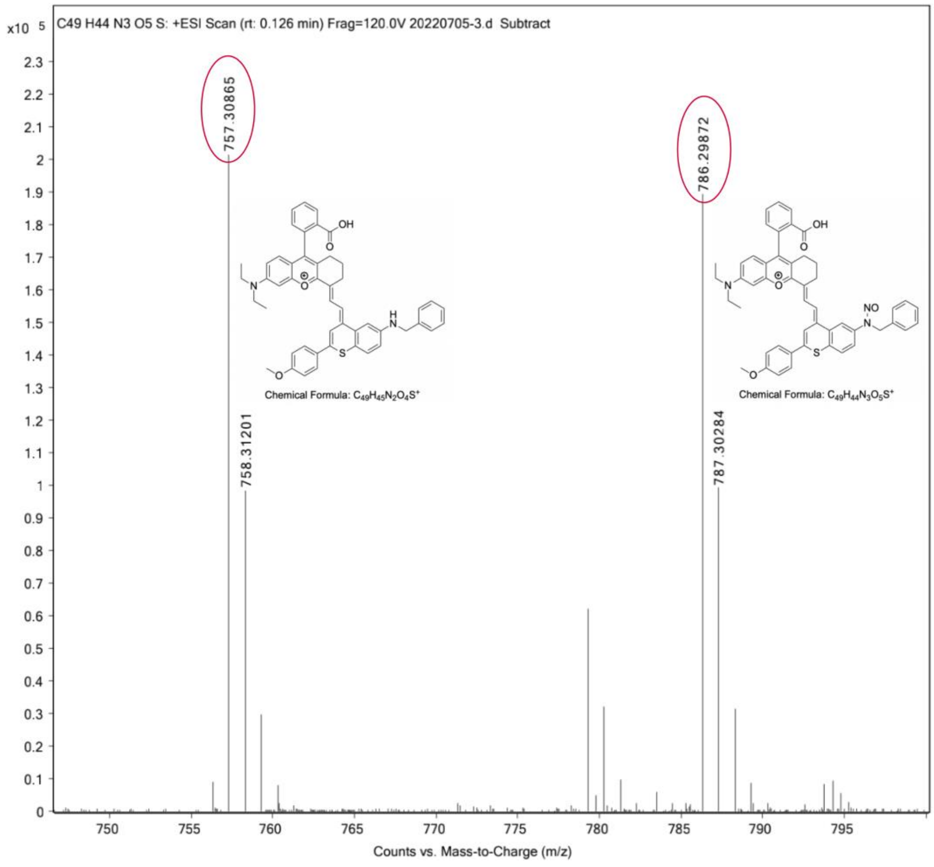

Figure S6. HRMS of HPLC monitored photo-release of RBT2-NO.

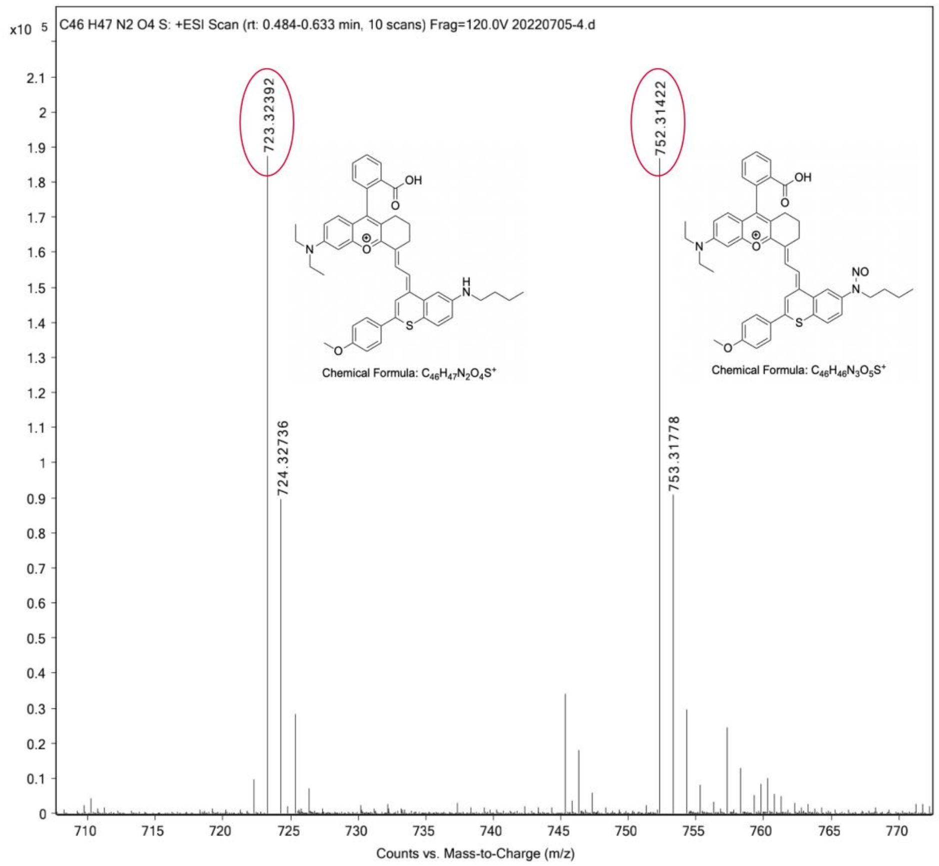

Figure S7. HRMS of HPLC monitored photo-release of RBT3-NO.

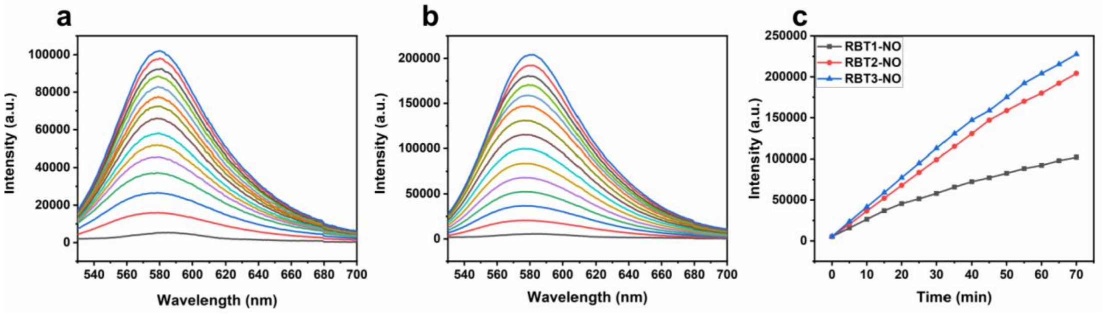

Figure S8. NO releasing verification of RBT1-NO (a) and RBT2-NO (b) with RhBs, and comparison of fluorescence emission spectrum changes of RBT-NO (c).

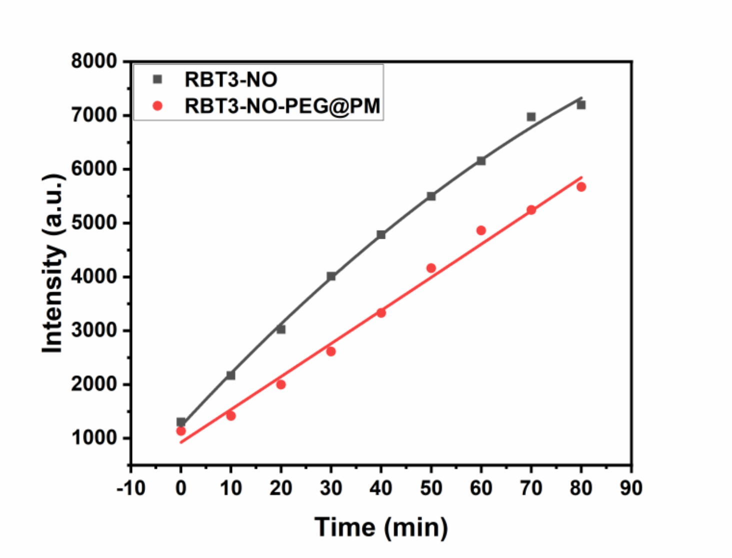

Figure S9. Comparison of fluorescence emission spectrum changes of RBT3-NO-PEG@PM (10 μM, calculated as RBT3-NO) and RBT3-NO (10 μM) irradiated by 808 nm laser at a fluence rate of 0.5 W cm^{-2}.

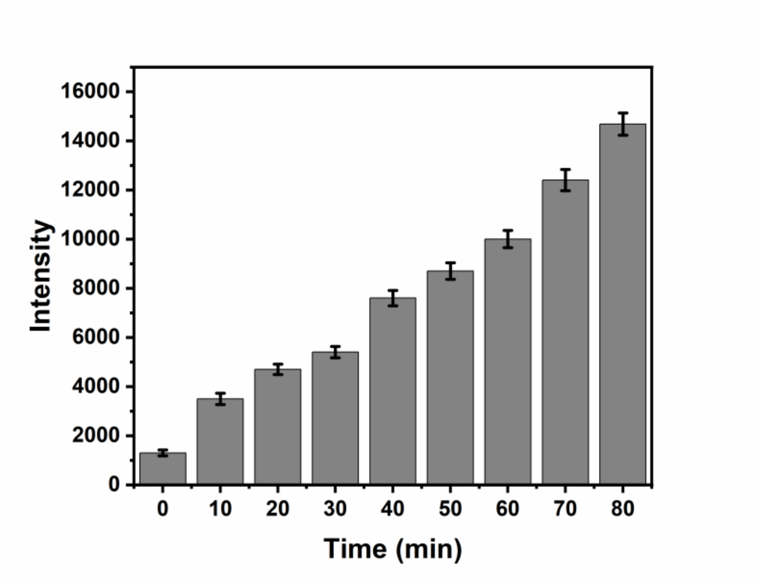

Figure S10. Quantitative analysis of fluorescence intensity at different times.

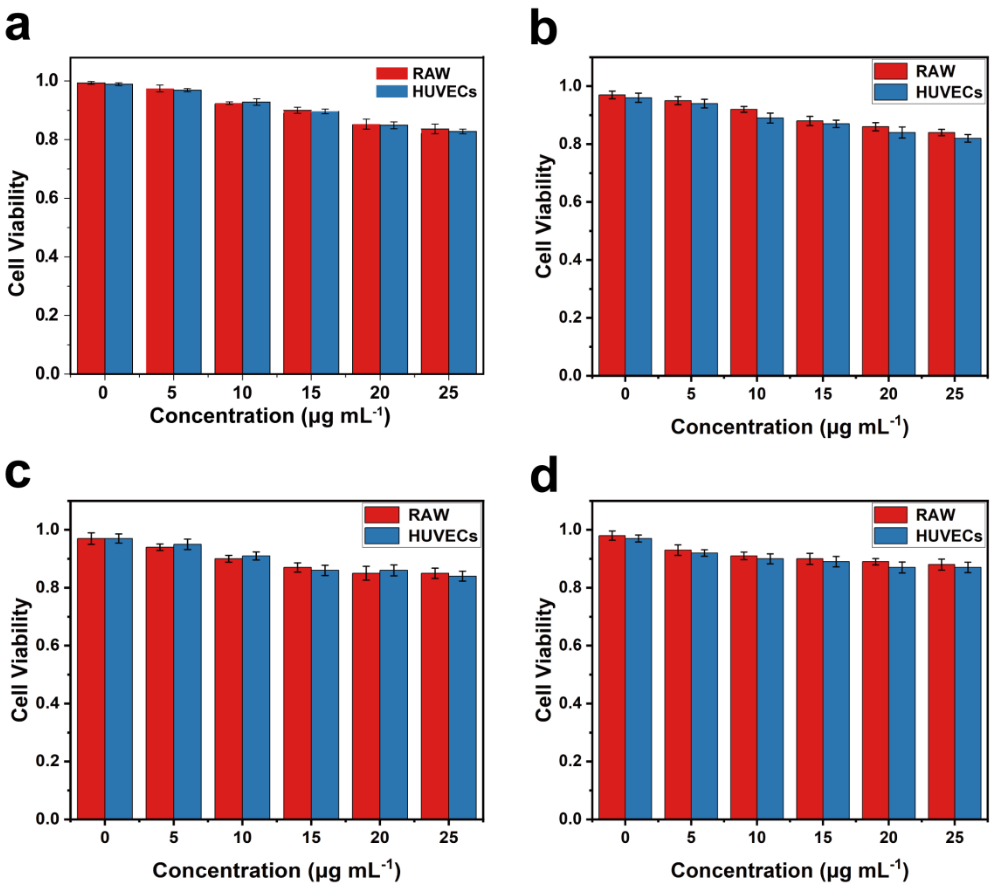

Figure S11. (a) MTT assay of RBT3-NO-PEG@PM co-cultured for 24 hours in RAW cells and HUVECs cells. (b) MTT assay of RBT3-NO-PEG@PM co-cultured for 48 hours in RAW cells and HUVECs cells. (c) MTT assay of RBT3-NO co-cultured for 24 hours in RAW cells and HUVECs cells. (d) MTT assay of RBT3 co-cultured for 24 hours in RAW cells and HUVECs cells.

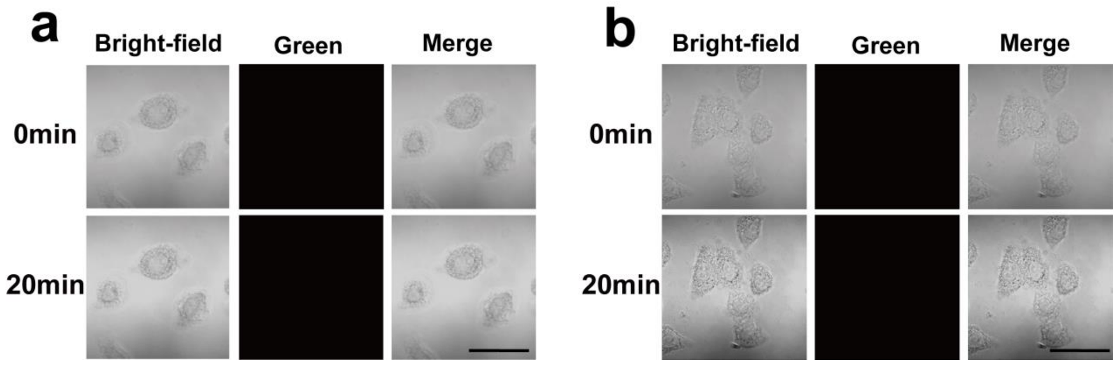

Figure S12. Fluorescence imaging of proving gaseous NO release in RAW and HUVECs cells co-incubated with RBT3-NO-PEG@PM (15 μg mL^{-1}) and NO indicator DAF-FM-DA (20 μM) with no laser exposure (scale bar: 20 μm).

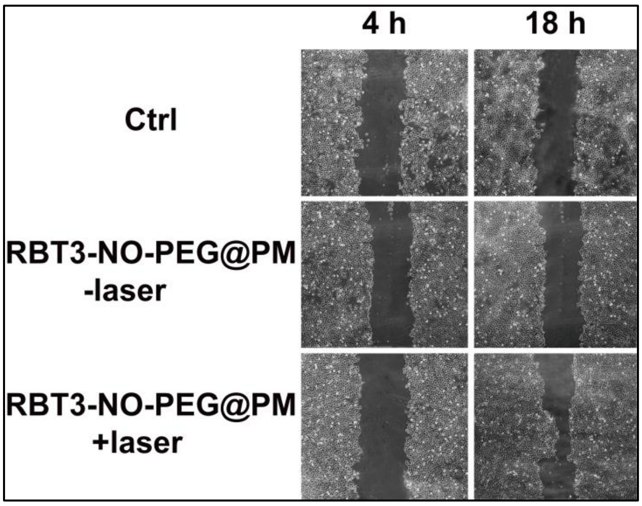

Figure S13. Validation of endothelial cell migration ability in HUVECs cells co-incubated with RBT3-NO-PEG@PM (15 μg mL^{-1}) with NIR light exposure (808 nm, 0.5 W cm^{-2}).

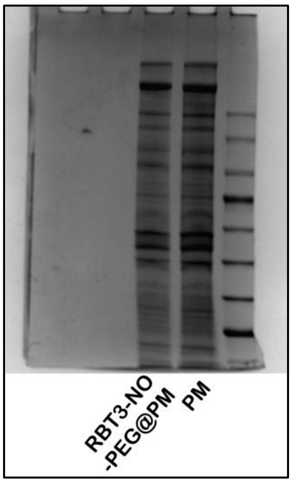

Figure S14. Protein content analysis of PM and RBT3-NO-PEG@PM using Coomassie blue staining.

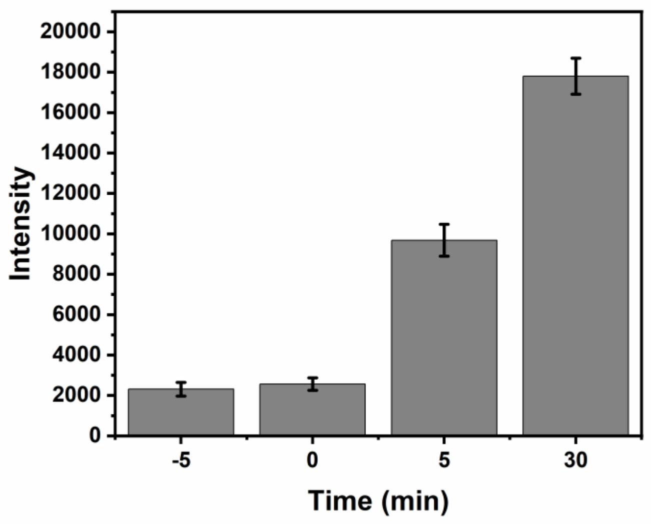

Figure S15. Quantitative analysis of fluorescence intensity at different times.

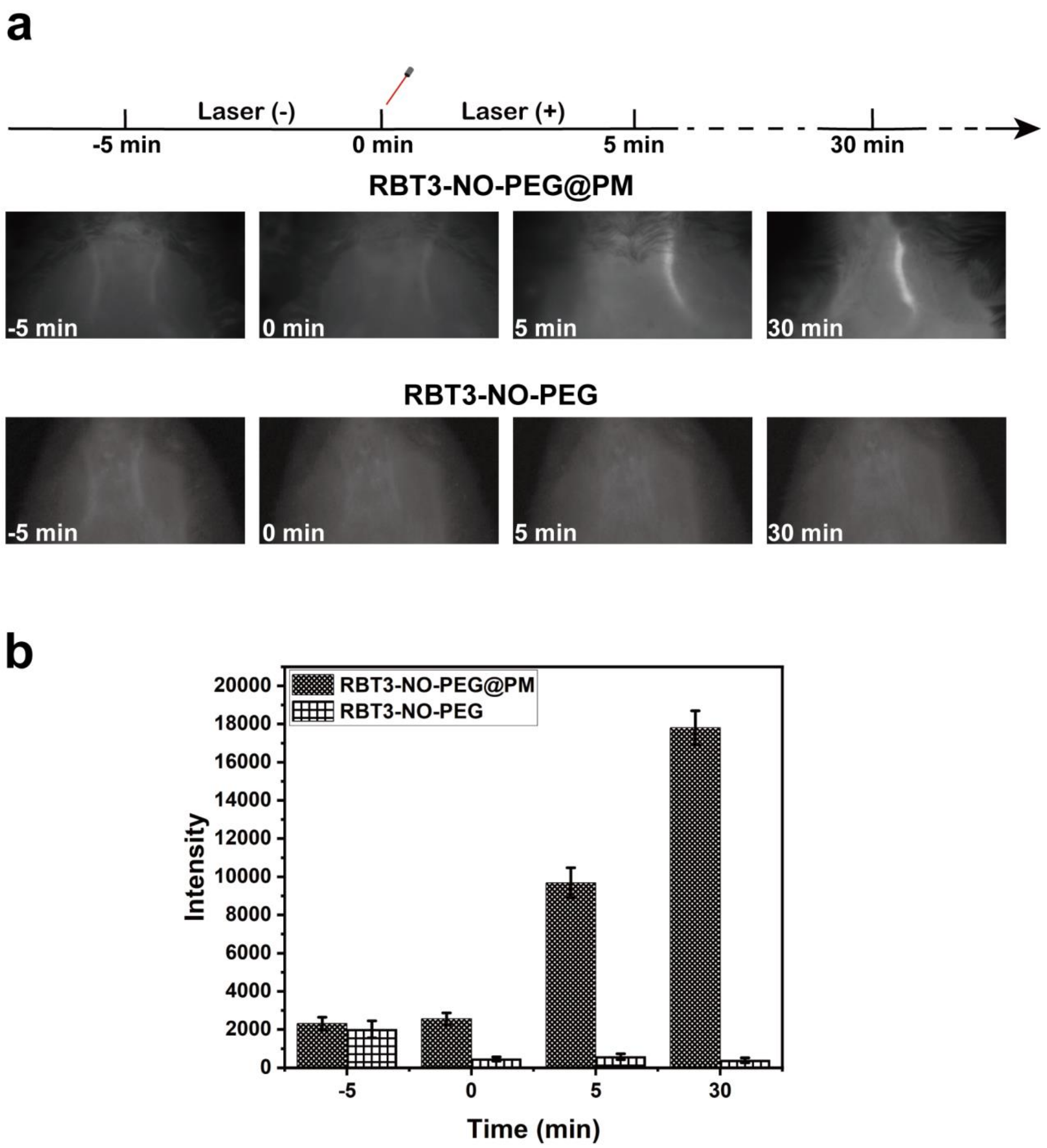

Figure S16. (a) NIR-II Fluorescence image of RBT3-NO-PEG@PM and RBT3-NO-PEG arriving at the plaque with blood circulation after injection through the tail vein of mice. After 5 minutes, the plaque was irradiated with an 808 nm laser at a fluence rate of 0.5 W cm^{-2}. (b) Quantitative analysis of fluorescence intensity at different times.

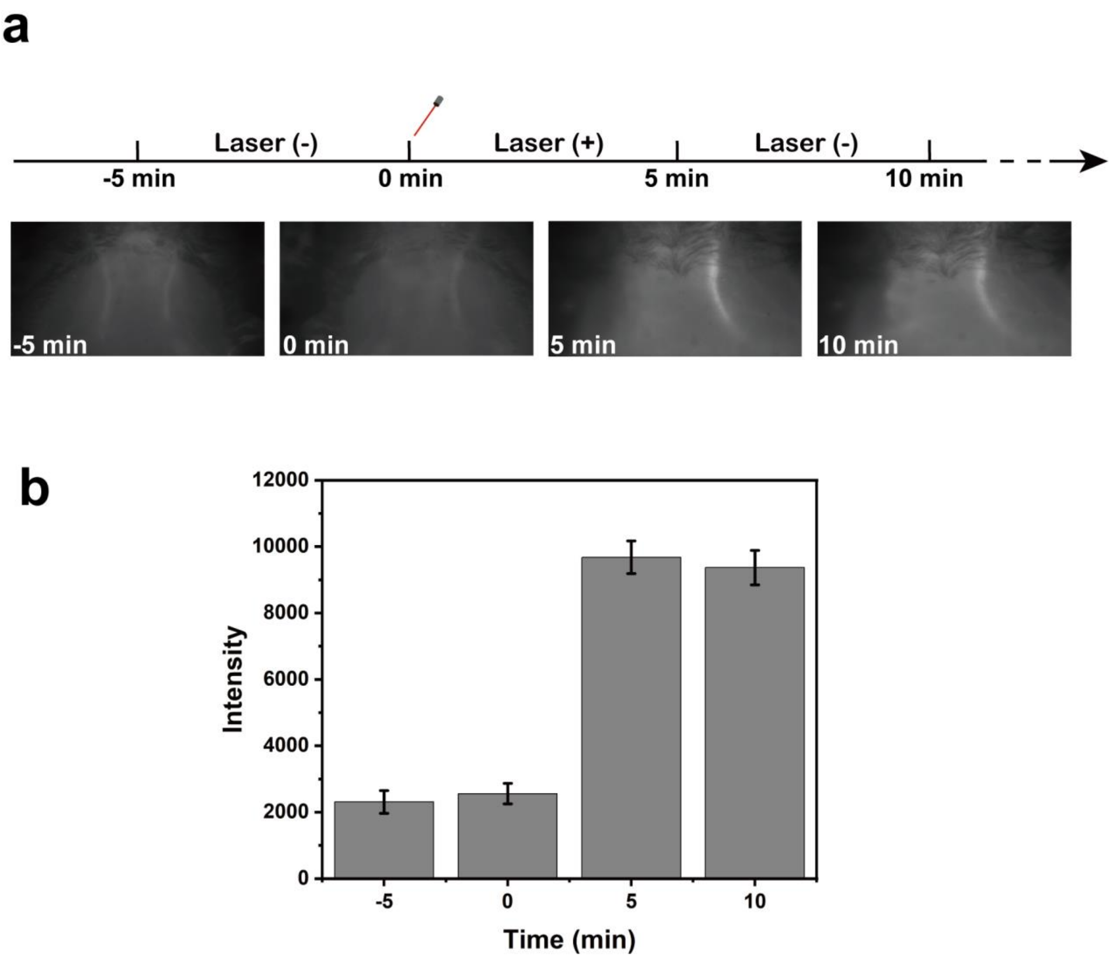

Figure S17. (a) NIR-II Fluorescence image of RBT3-NO-PEG@PM arriving at the plaque with blood circulation after injection through the tail vein of mice. After 5 minutes, the plaque was irradiated with an 808 nm laser at a fluence rate of 0.5 W cm^{-2}. (b) Quantitative analysis of fluorescence intensity at different times.

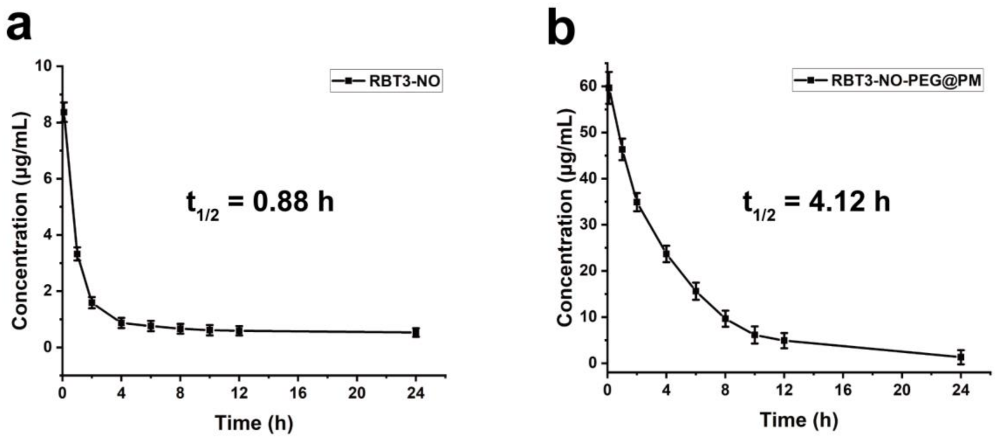

Figure S18. Pharmacokinetic curve and half-life of RBT3-NO and

RBT3-NO-PEG@PM after tail vein administration.

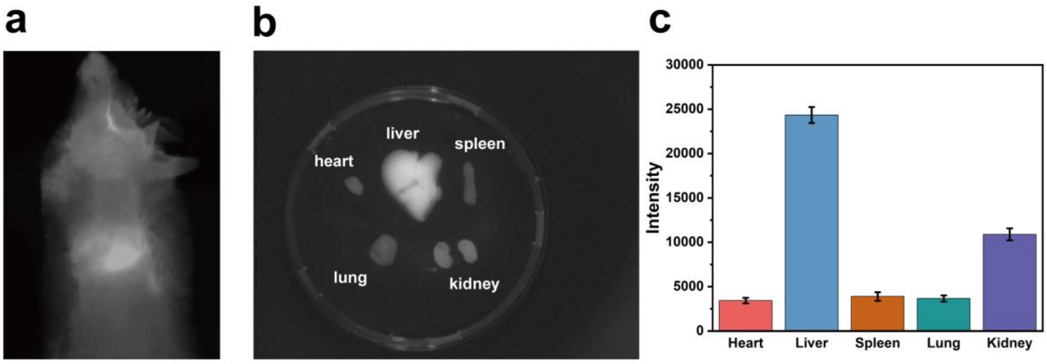

Figure S19. (a) NIR-II Fluorescence image of RBT3-NO-PEG@PM arriving at various tissue with blood circulation after injection through the tail vein of mice. After 5 minutes, the mice were irradiated with an 808 nm laser at a fluence rate of 0.5 W cm^{-2} for 30 min. (b) Tissue distribution map of RBT3-NO-PEG@PM in the main organs of mice. After 4 hours of injection into the tail vein, the main organs *in vitro* were irradiated with an 808 nm laser at a fluence rate of 0.5 W cm^{-2} for 30 min. (c) Quantitative analysis of fluorescence intensity of major organs.

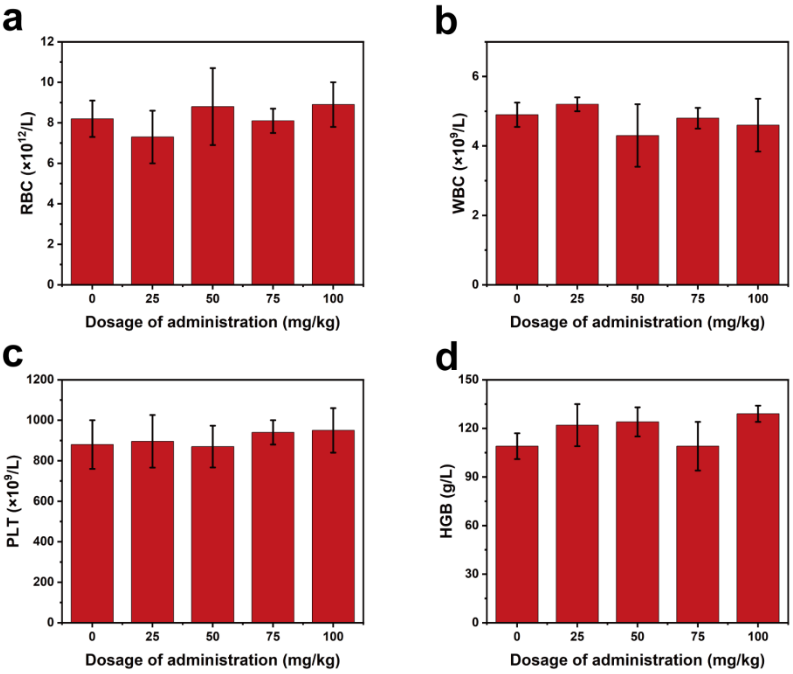

Figure S20. Whole blood cell count analysis in treatment groups with different doses of medication (RBC: red blood cell; WBC: white blood cell; PLT: platelet; HGB: Hemoglobin).

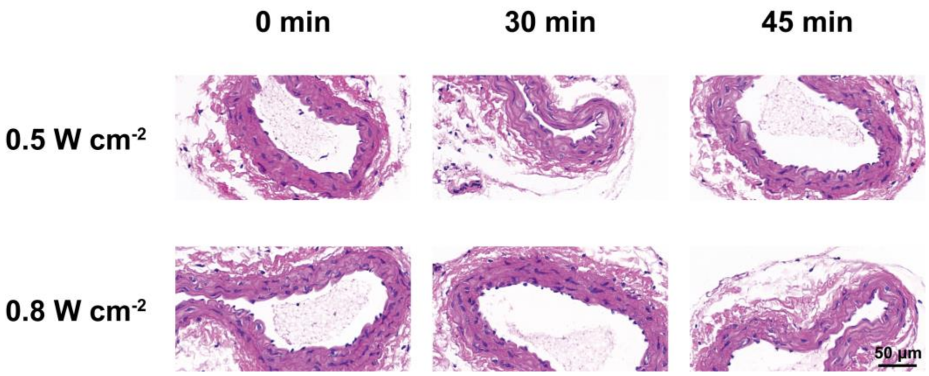

Figure S21. H&E stain images of blood vessels using different laser strengths (0.5 and 0.8 W cm^{-2}) for different irradiation times (0, 30, and 45 min).

11. Structural Characterization

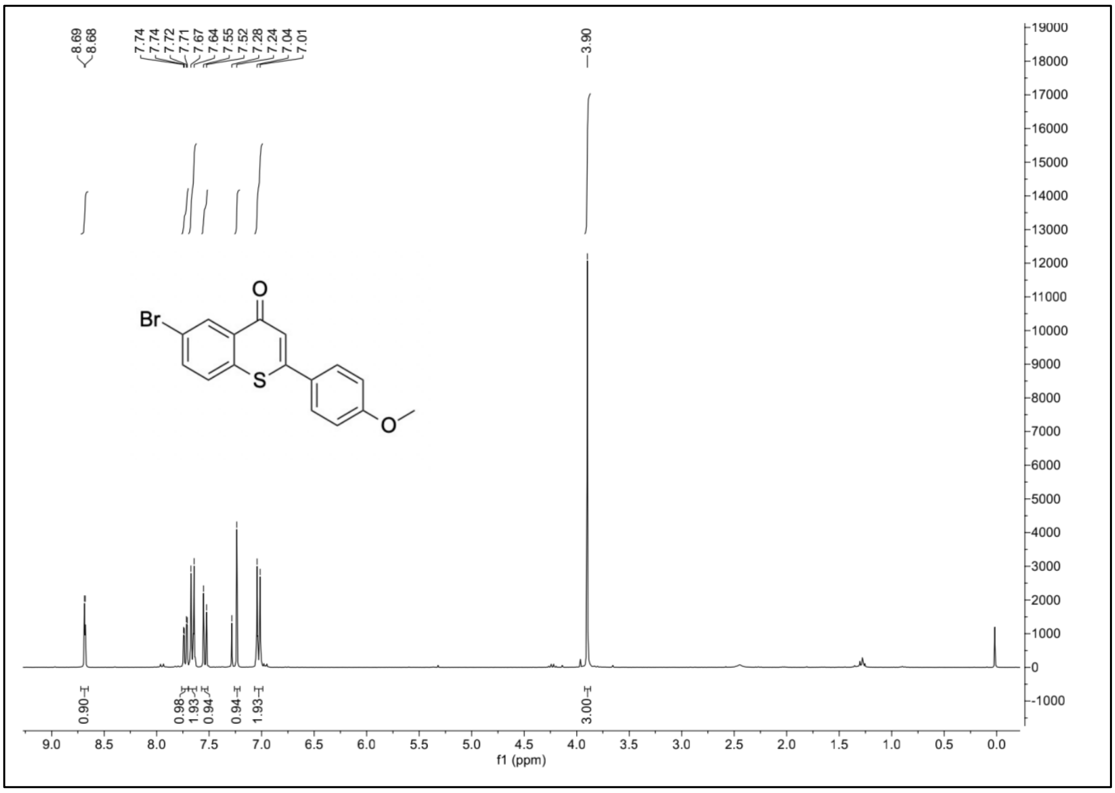

Figure S22. ^{1}H NMR spectrum of compound 3 in $CDCl_3$.

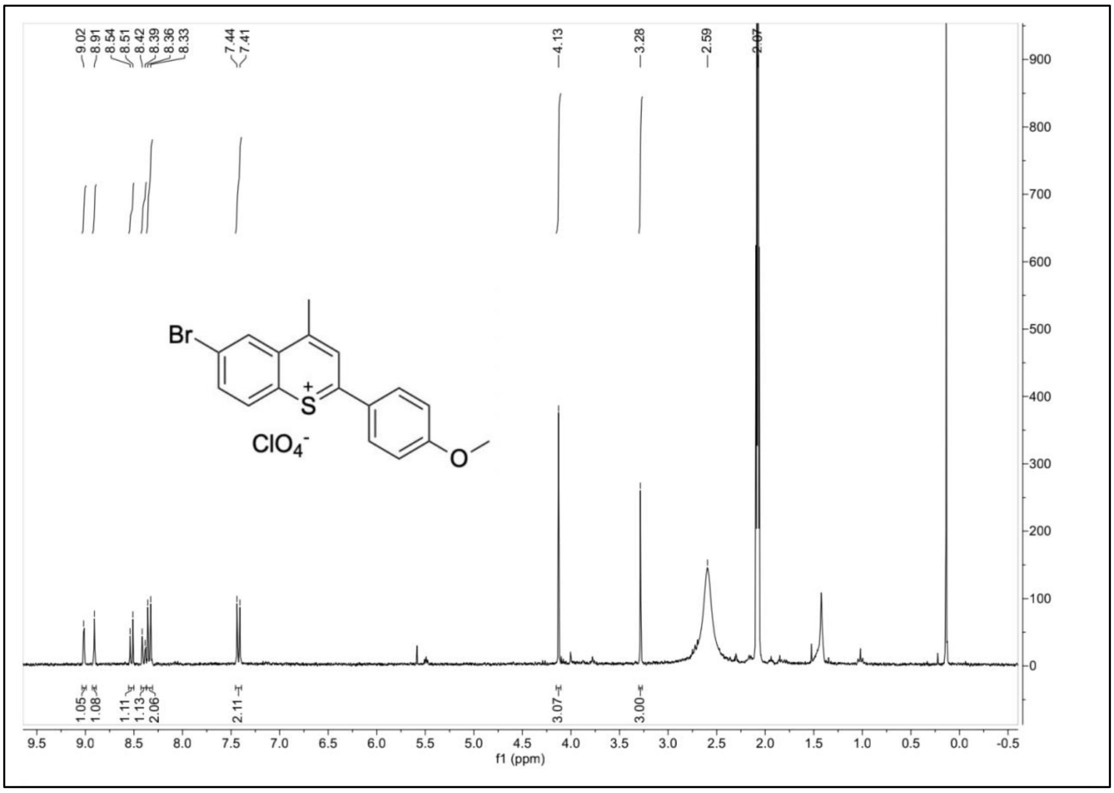

Figure S23. ^{1}H NMR spectrum of compound 4 in CD_3CN.

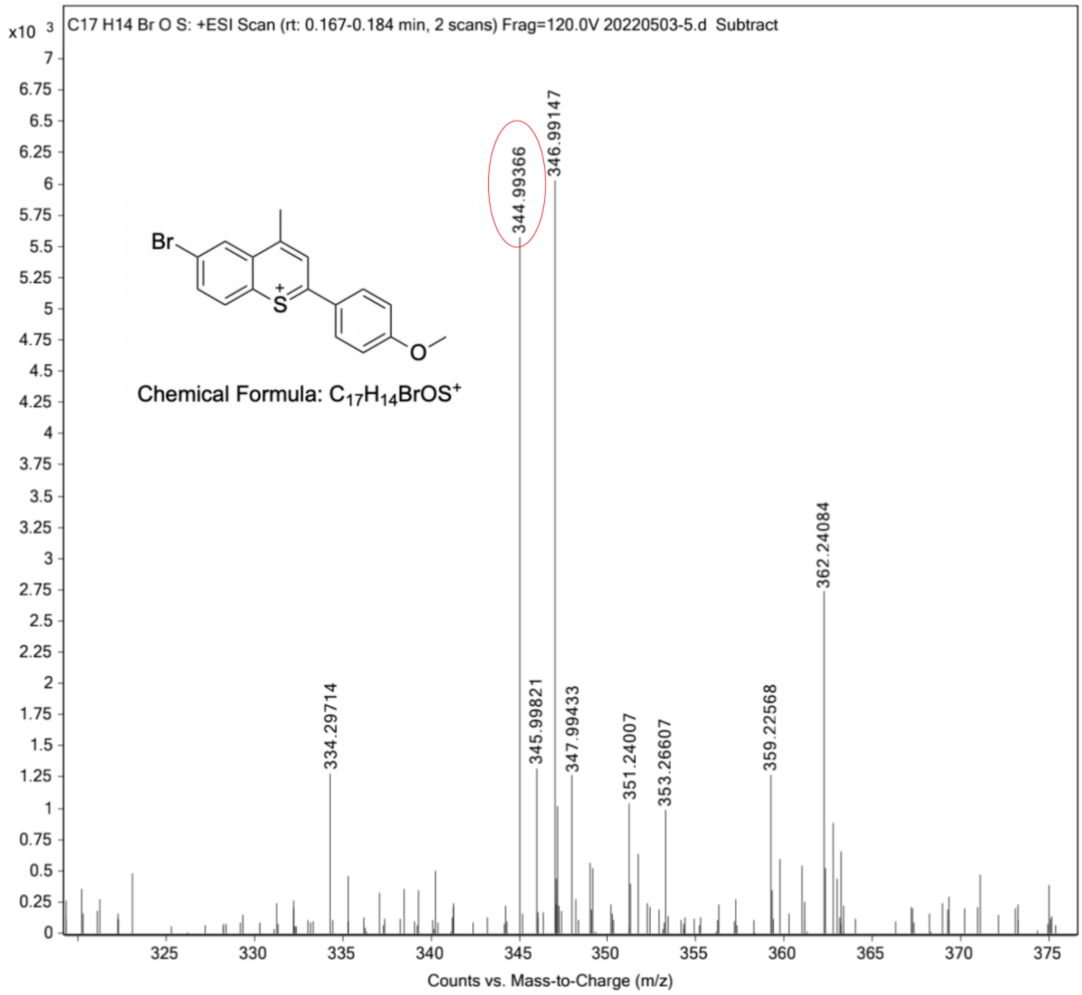

Figure S24. HRMS spectrum of compound 4.

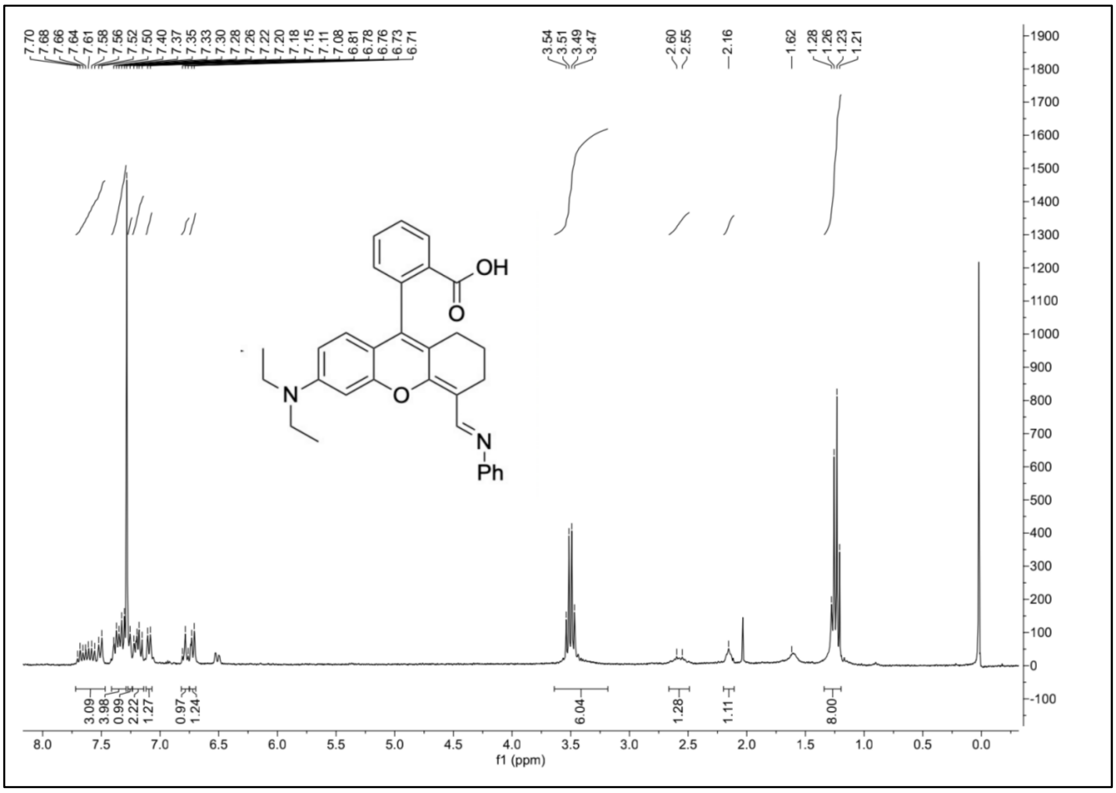

Figure S25. ^{1}H NMR spectrum of compound 7 in $CDCl_3$.

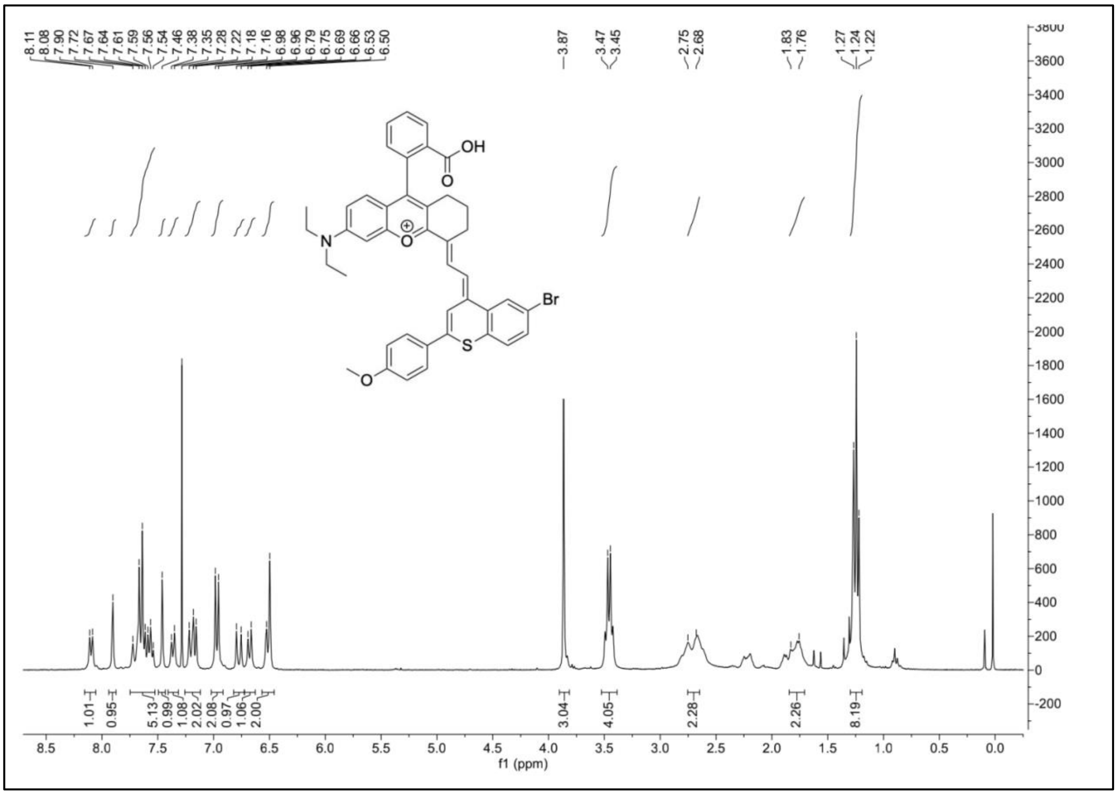

Figure S26. ^{1}H NMR spectrum of compound RBT-Br in $CDCl_3$.

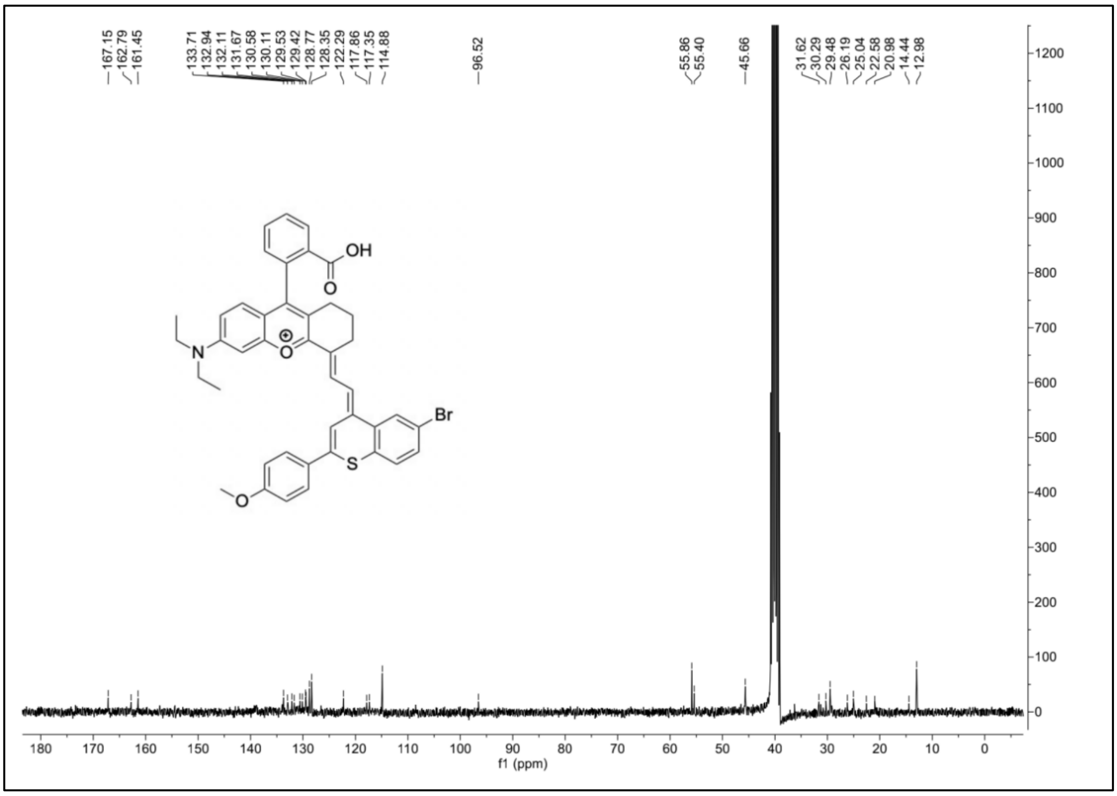

Figure S27. ^{13}C NMR spectrum of compound RBT-Br in DMSO-d_6.

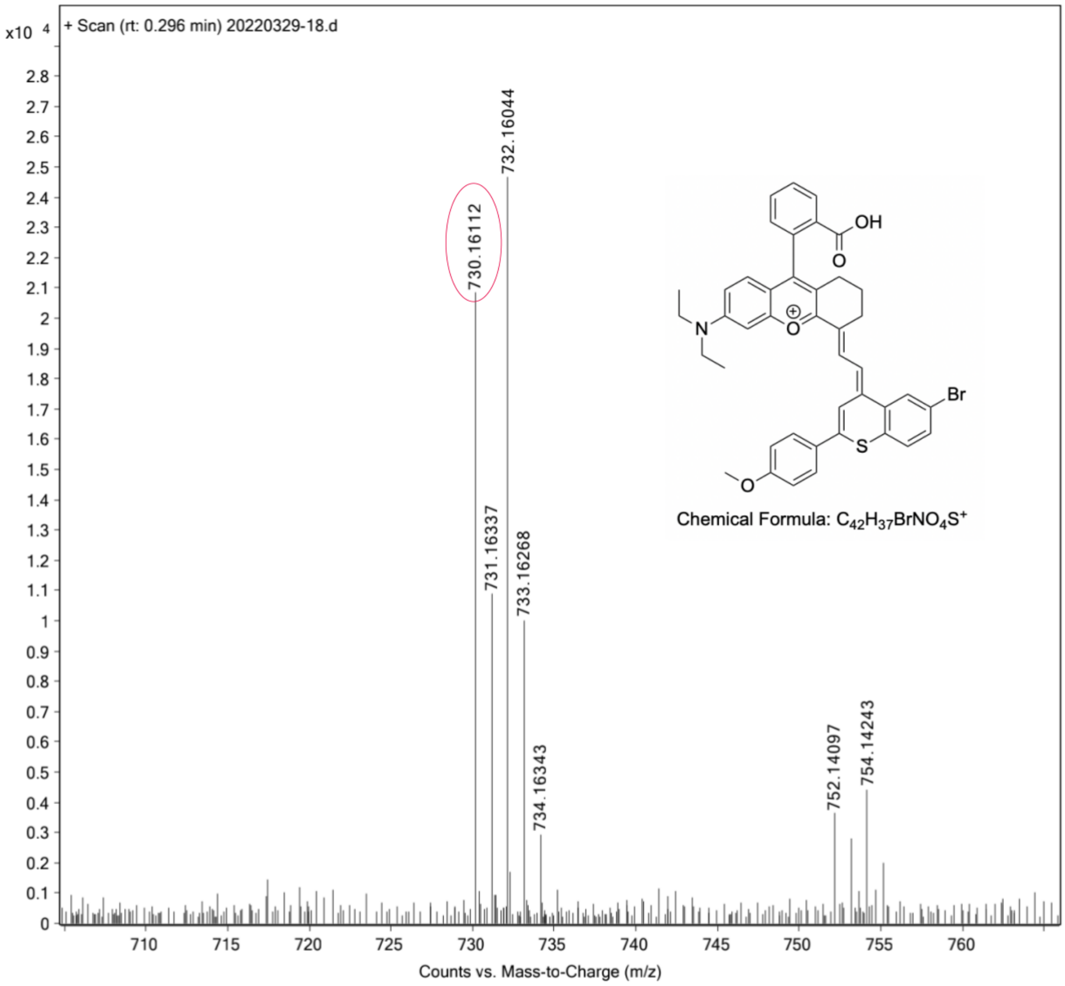

Figure S28. HRMS spectrum of compound RBT-Br.

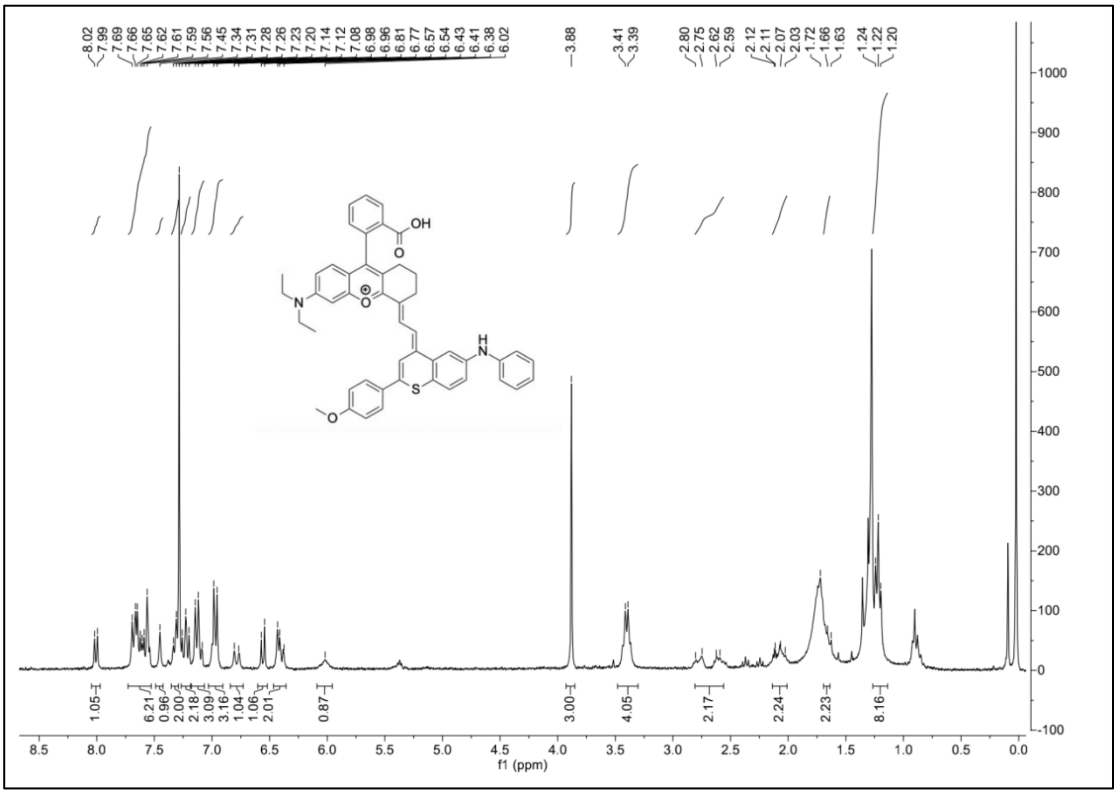

Figure S29. ^{1}H NMR spectrum of compound RBT1 in $CDCl_3$.

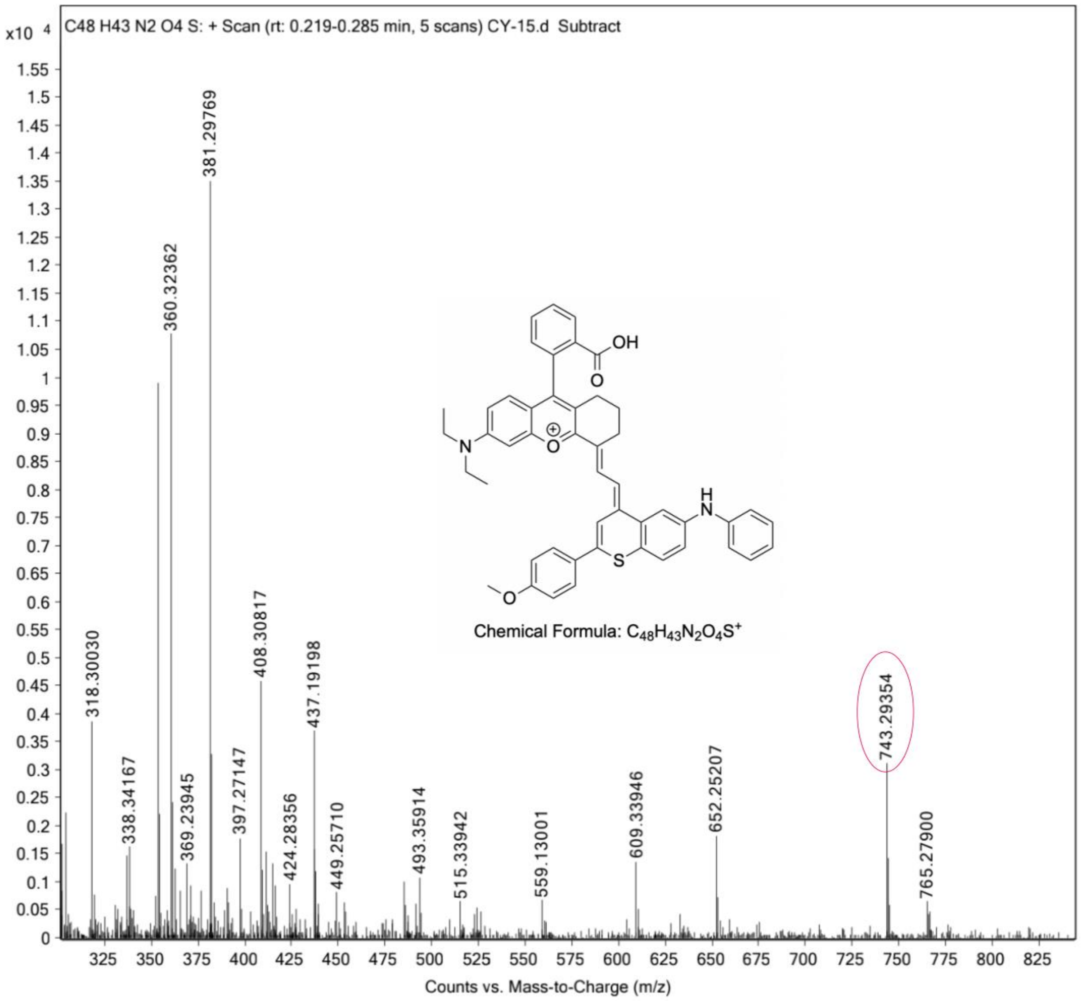

Figure S30. HRMS spectrum of compound RBT1.

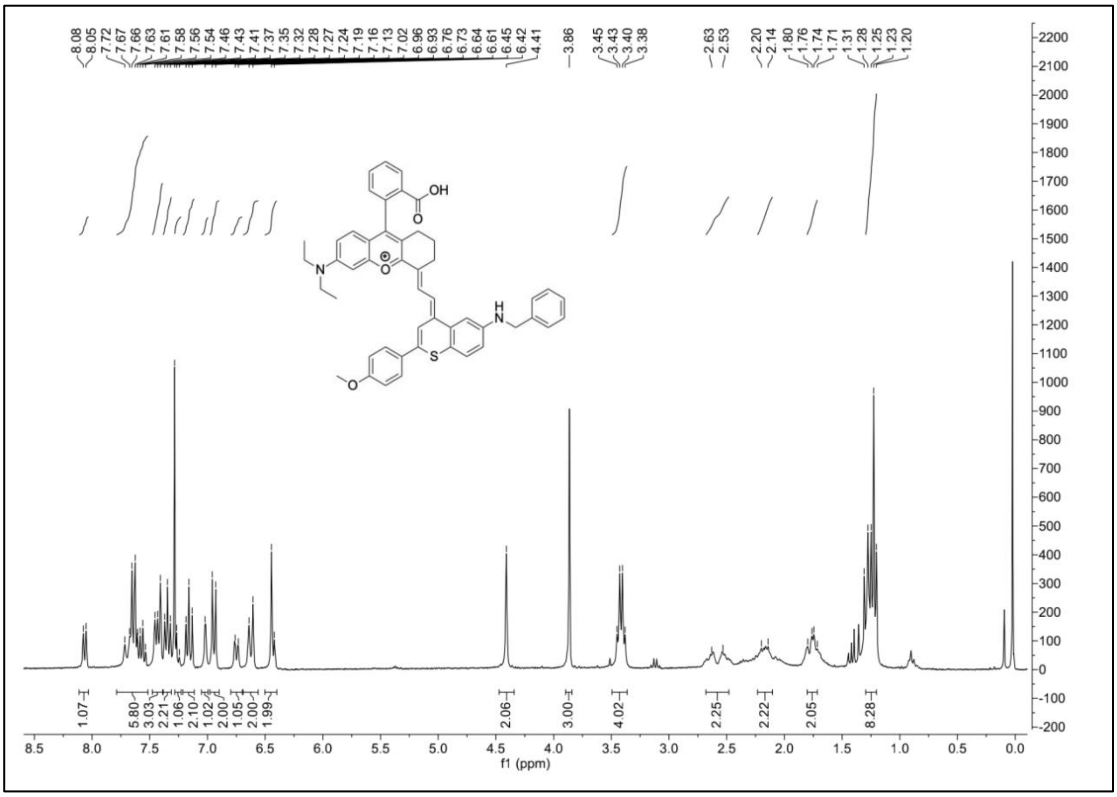

Figure S31. ^{1}H NMR spectrum of compound RBT2 in $CDCl_3$.

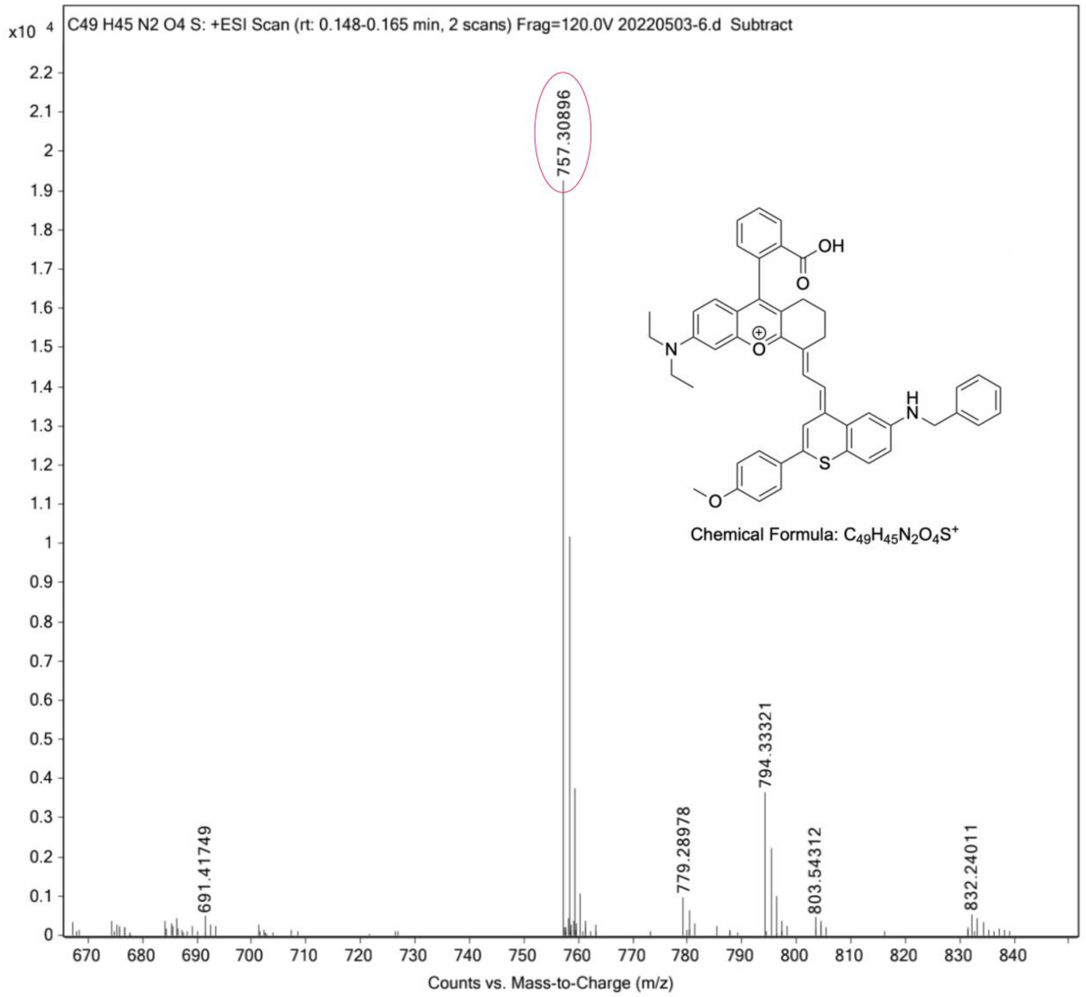

Figure S32. HRMS spectrum of compound RBT2.

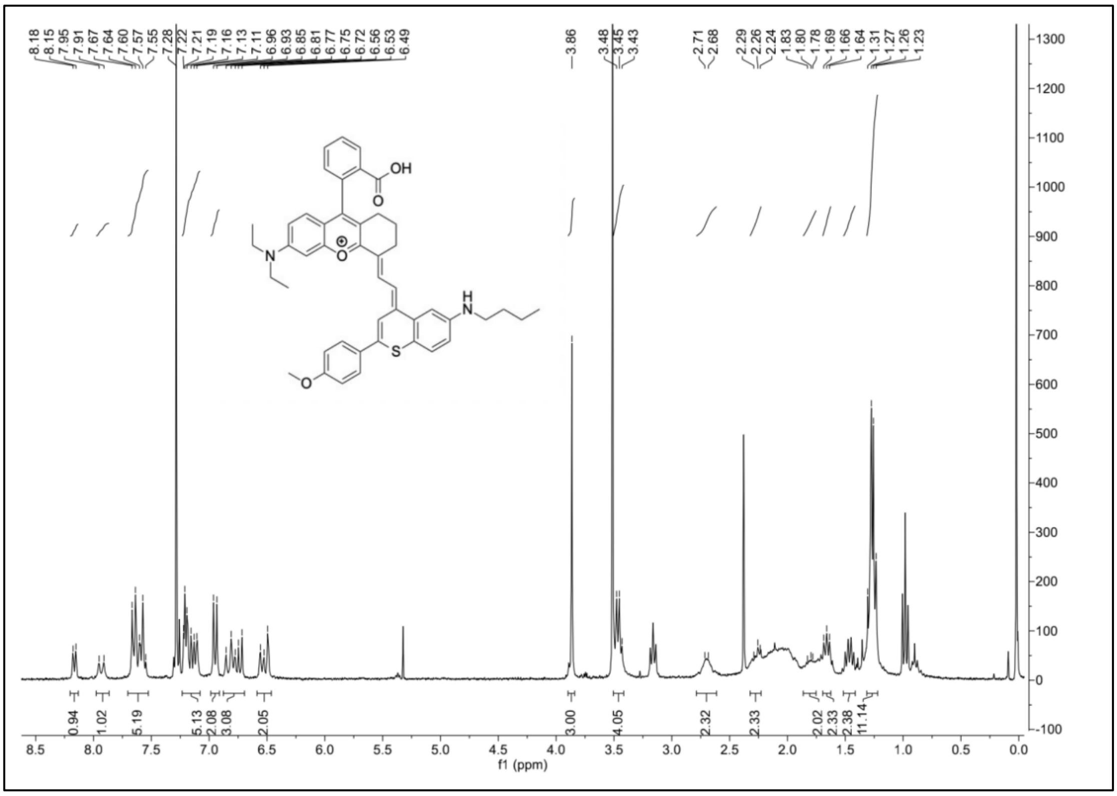

Figure S33. ^{1}H NMR spectrum of compound RBT3 in $CDCl_3$.

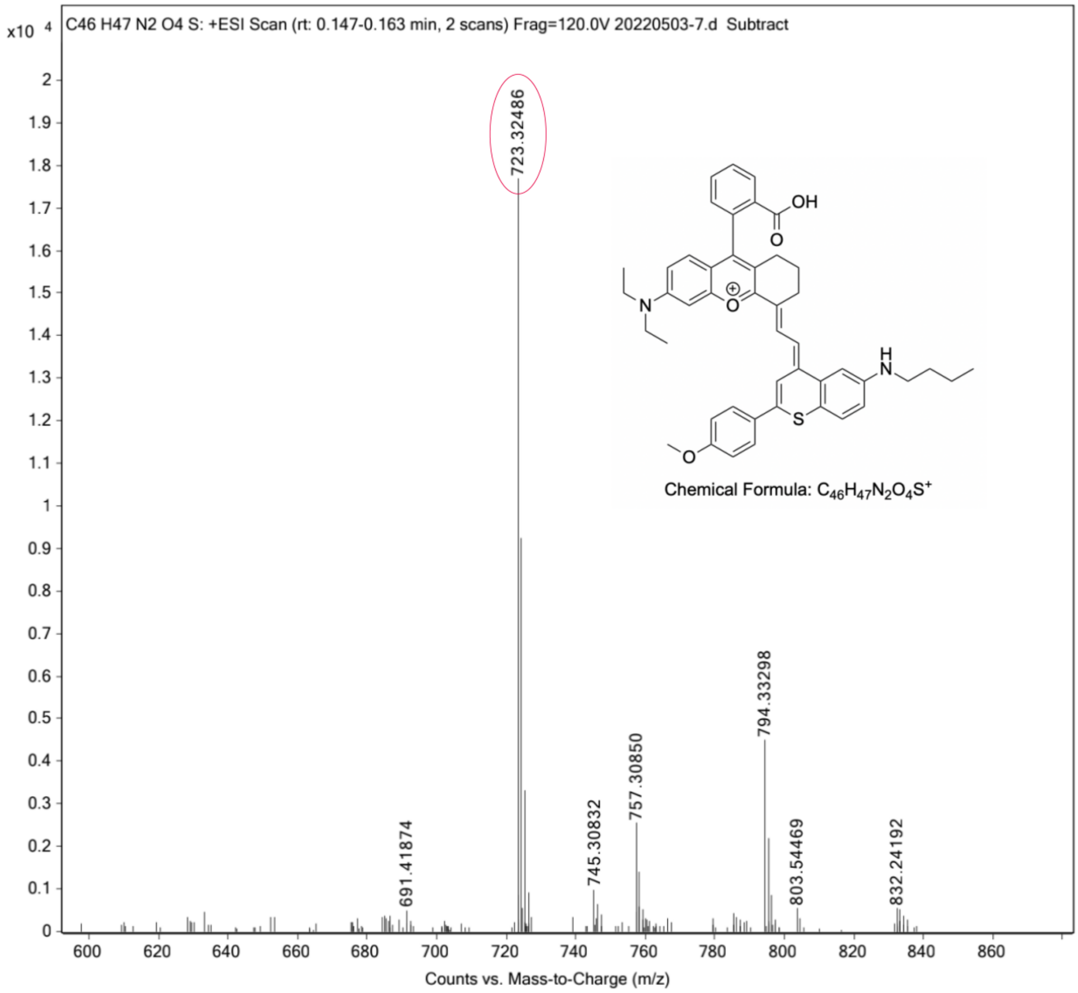

Figure S34. HRMS spectrum of compound RBT3.

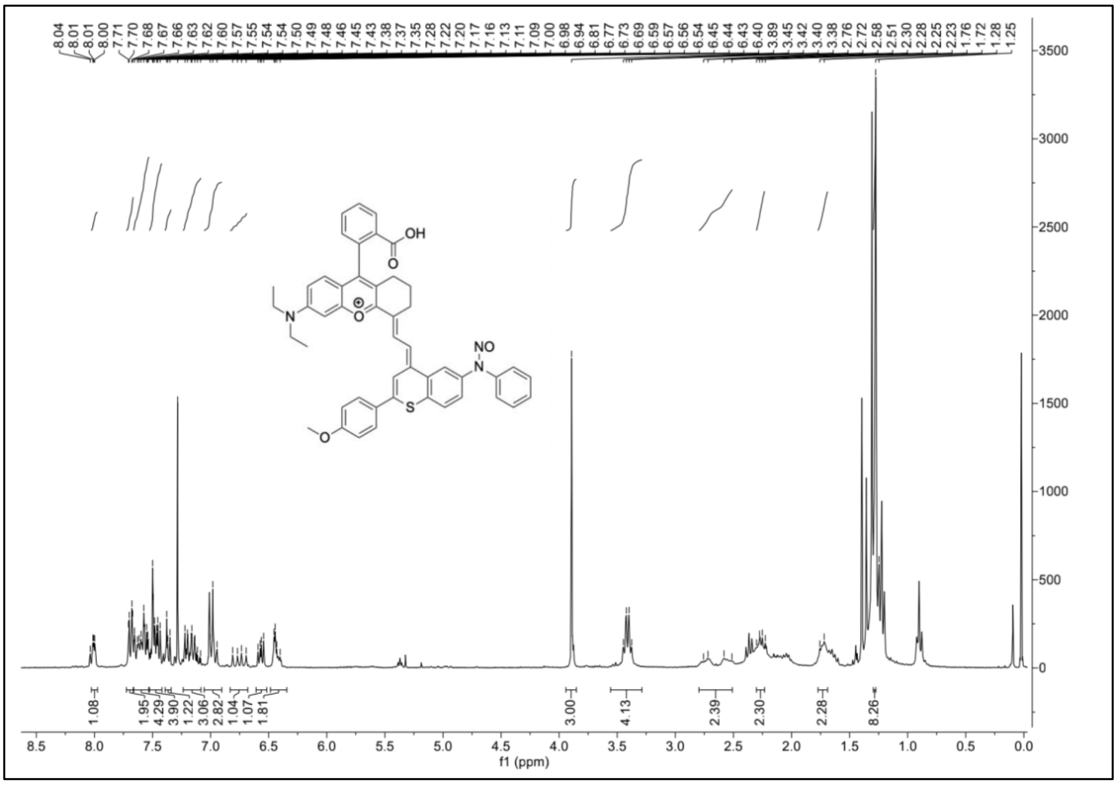

Figure S35. ^{1}H NMR spectrum of compound RBT1-NO in $CDCl_3$.

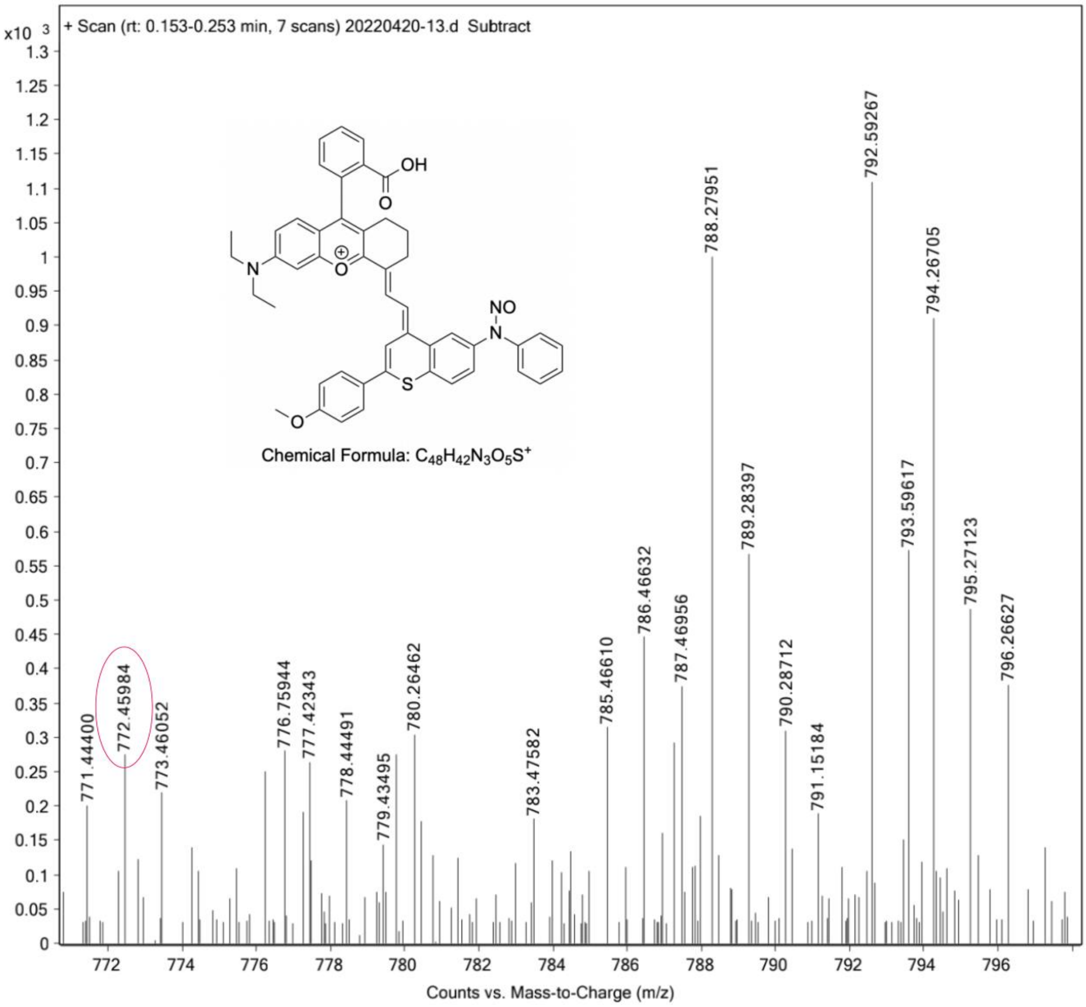

Figure S36. HRMS spectrum of compound RBT1-NO.

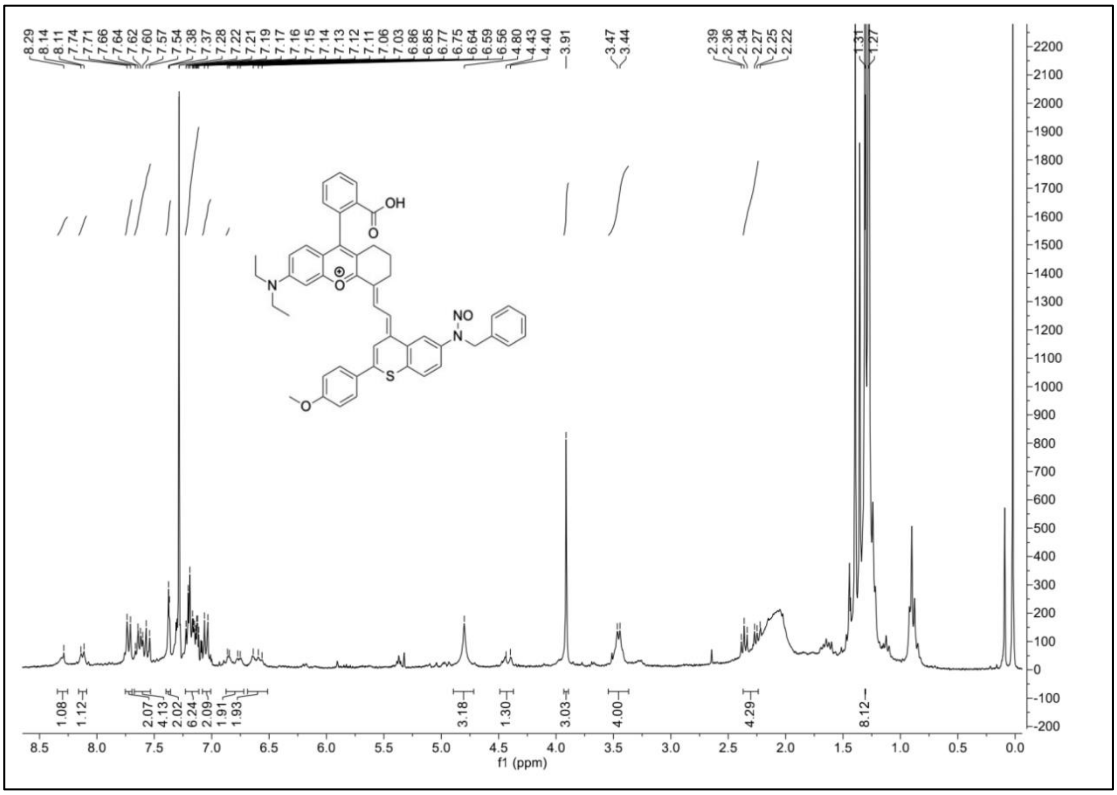

Figure S37. ^{1}H NMR spectrum of compound RBT2-NO in $CDCl_3$.

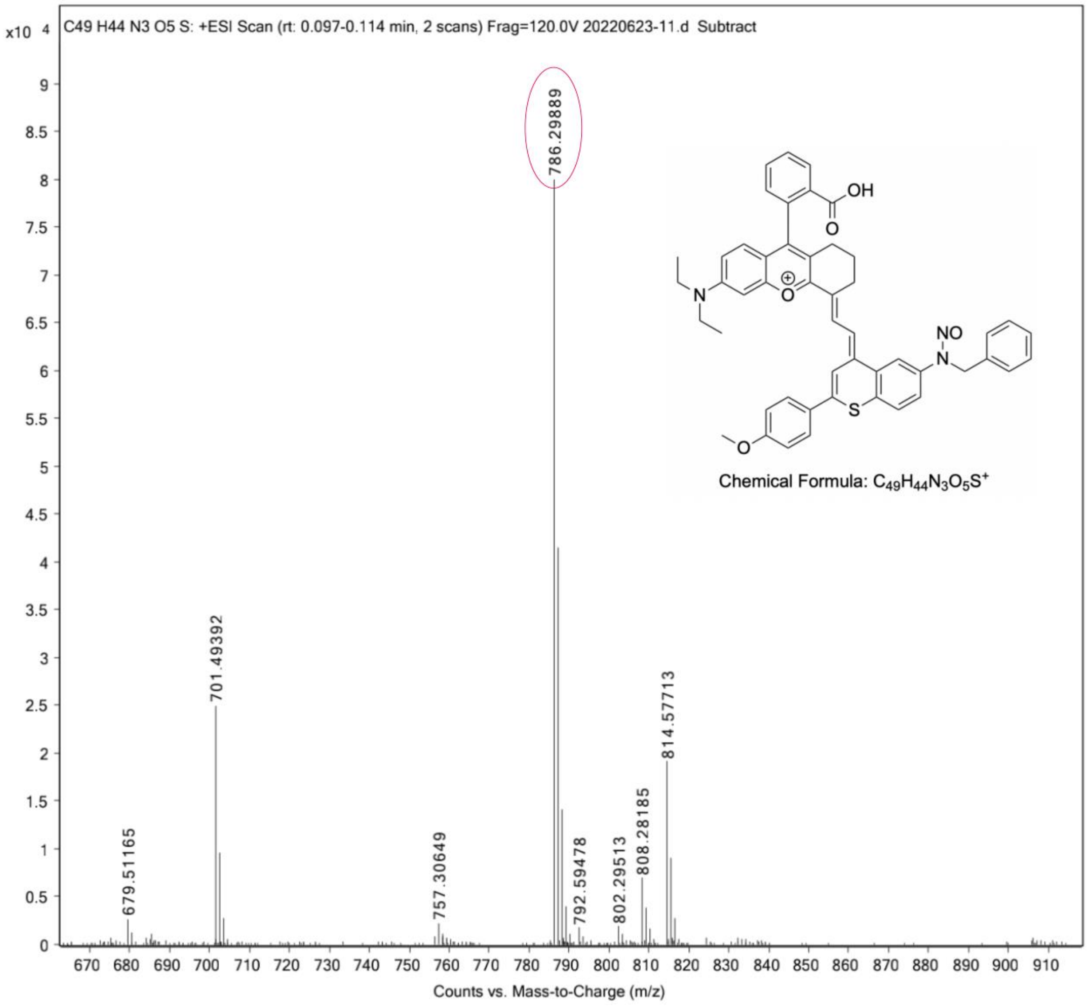

Figure S38. HRMS spectrum of compound RBT2-NO.

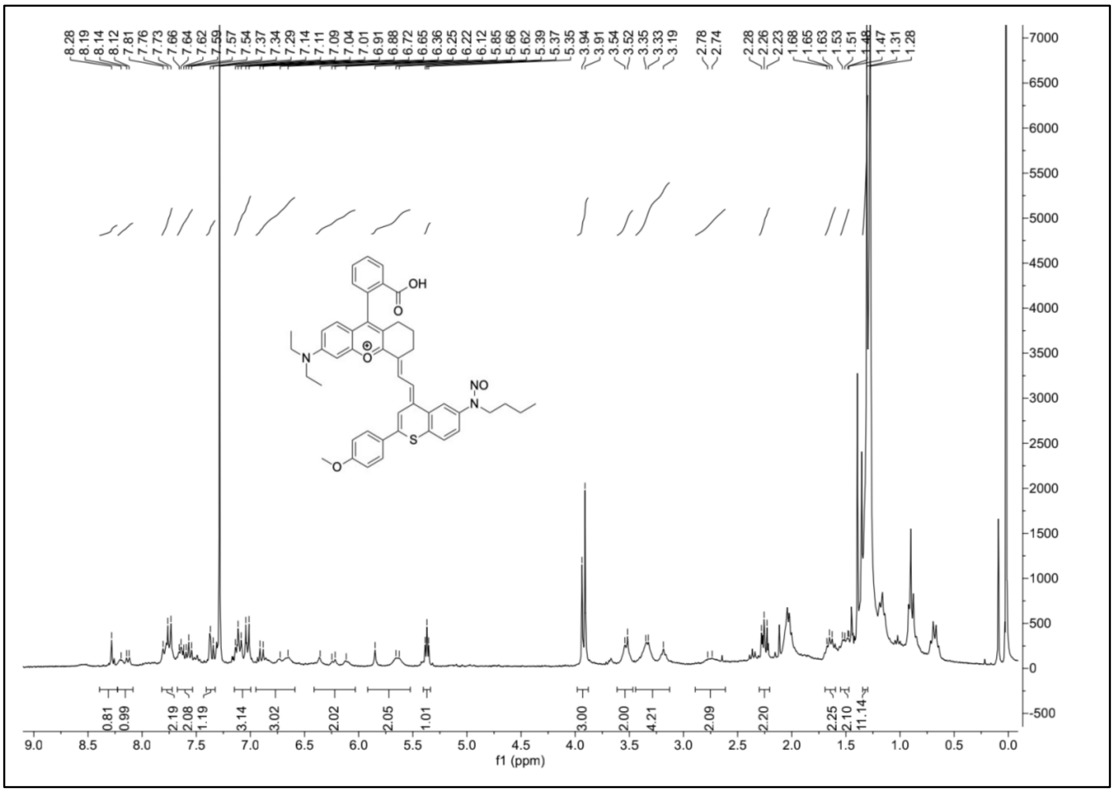

Figure S39. ^{1}H NMR spectrum of compound RBT3-NO in $CDCl_3$.

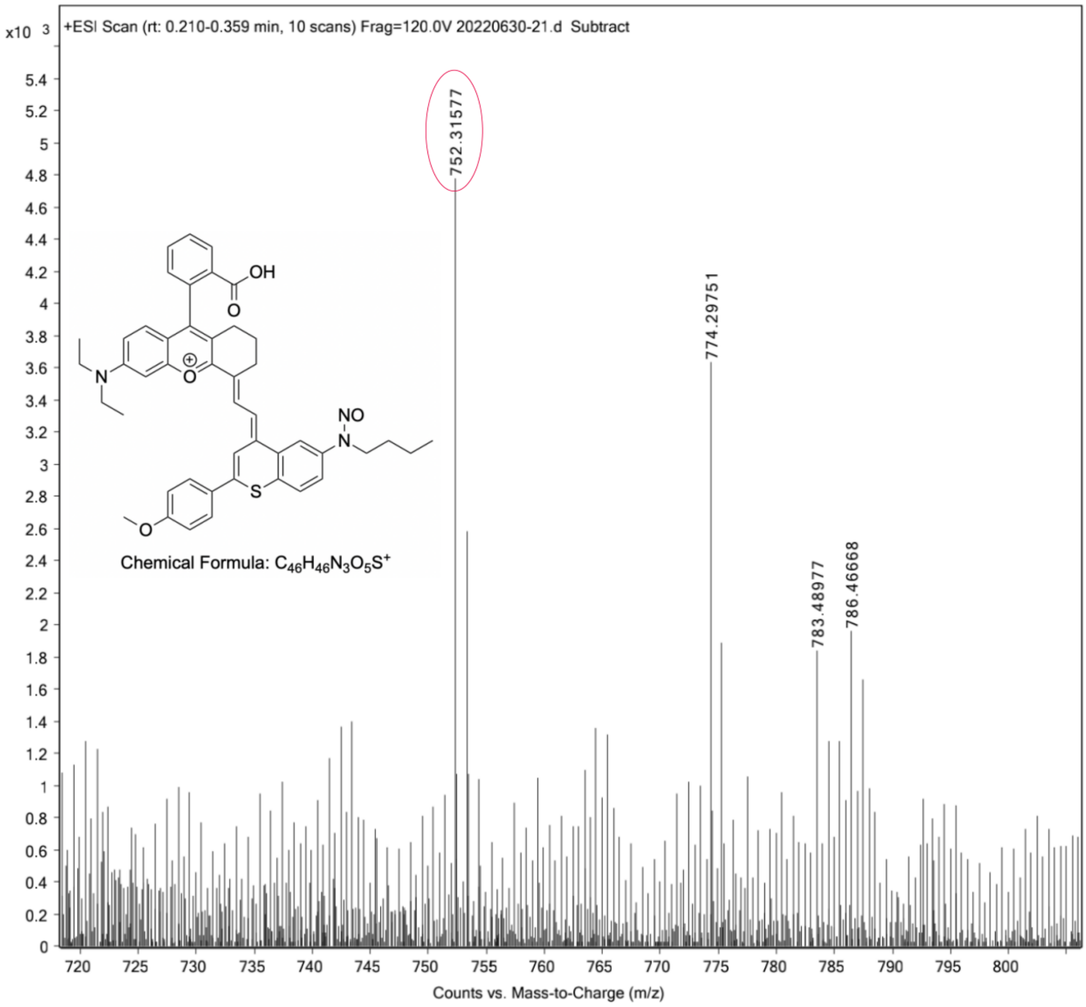

Figure S40. HRMS spectrum of compound RBT3-NO.

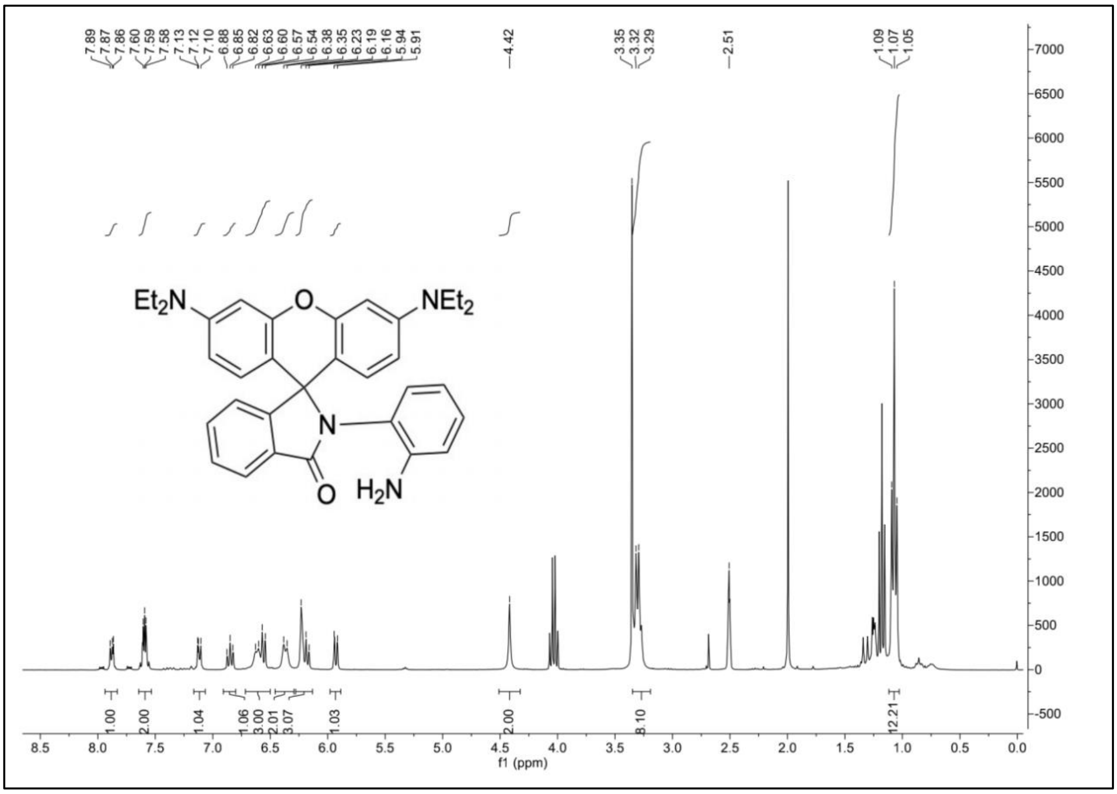

Figure S41. ¹H NMR spectrum of compound RhBs in DMSO-d_6.
